# An efficient methodological approach for synthesis of selenopyridines: generation, reactions, anticancer activity, EGFR inhibitory activity and molecular docking studies

**DOI:** 10.1007/s11030-024-10872-2

**Published:** 2024-05-13

**Authors:** Bahgat R. M. Hussein, Sham M. M. El-Saghier, Rasha M. Allam, Mamdouh F. A. Mohamed, Amer A. Amer

**Affiliations:** 1 Department of Chemistry, Faculty of Science, Sohag University, Sohag, 82524 Egypt; 2 Pharmacology Department, National Research Centre, Giza, 11865 Egypt; 3 Department of Pharmaceutical Chemistry, Faculty of Pharmacy, Sohag University, Sohag, 82524 Egypt

**Keywords:** Selenopyridine, Selenopheno[2,3-*b*]pyridine, Selenoazo dyes, Anticancer, EGFR inhibitor

## Abstract

In the present work, we successfully synthesized *Se*-alkyl selenopyridines **1** and **3**, selenopheno[2,3-*b*]pyridine **2**, and bis-selenopyridine **4** derivatives using an eco-friendly method by utilizing NaHSe instead of toxic hydrogen selenide. The effect of the temperature on the reaction was screening at various temperatures. The regiospecific reaction of selenopyridine **1** with bromine afforded an unexpected product 4,6-diamino-5-bromo-2-[(cyanomethyl)selenyl]-pyridine-3-carbonitrile (**5**), which was cyclized to selenopheno[2,3-*b*]pyridine (**7**) by refluxing in the presence of TEA. While its treatment with thiophenol and/or *p*-chlorothiophenol gave **8a**,** b**. On the other hand, its reaction with aminothiophenol afforded 2-(benzo[*d*]-thiazol-2-yl)-5-bromoselenopheno[2,3-*b*]pyridine-3,4,6-triamine (**9**). Also, *N*-(2-cyano-4-methyl-5*H*-1-seleno-3,5,8-triazaacenaphthylen-7-yl)acetamide (**11**) and a novel series of selenoazo dyes **12a**–**d** were synthesized by treatment of selenopheno[2,3-*b*]pyridine **2** with acetic anhydride and/or diazonium chlorides of aromatic amines, respectively. Then, we ascertained the potential activity of synthesized compounds against highly metastatic prostate cancer cells (PC-3) and osteosarcoma cells (MG-63) and found that **12a**, **12b**, **12c**, and **12d** were more cytotoxic than doxorubicin in both tested cell lines, showing nearly the same anticancer activity with IC_50_ values ranging from 2.59 ± 0.02 µM to 3.93 ± 0.23 µM. Mechanistically, the most potent compounds **12a** and **12b** proved to be potent EGFR inhibitors with IC_50_ values of 0.301 and 0.123 µM, respectively, compared to lapatinib as a positive reference (IC_50_ = 0.049 µM). Moreover, the docking results are in good agreement with the anticancer activity as well as the EGFR inhibitory activity, suggesting these two compounds as promising EGFR anticancer candidates.

**Graphical abstract:**

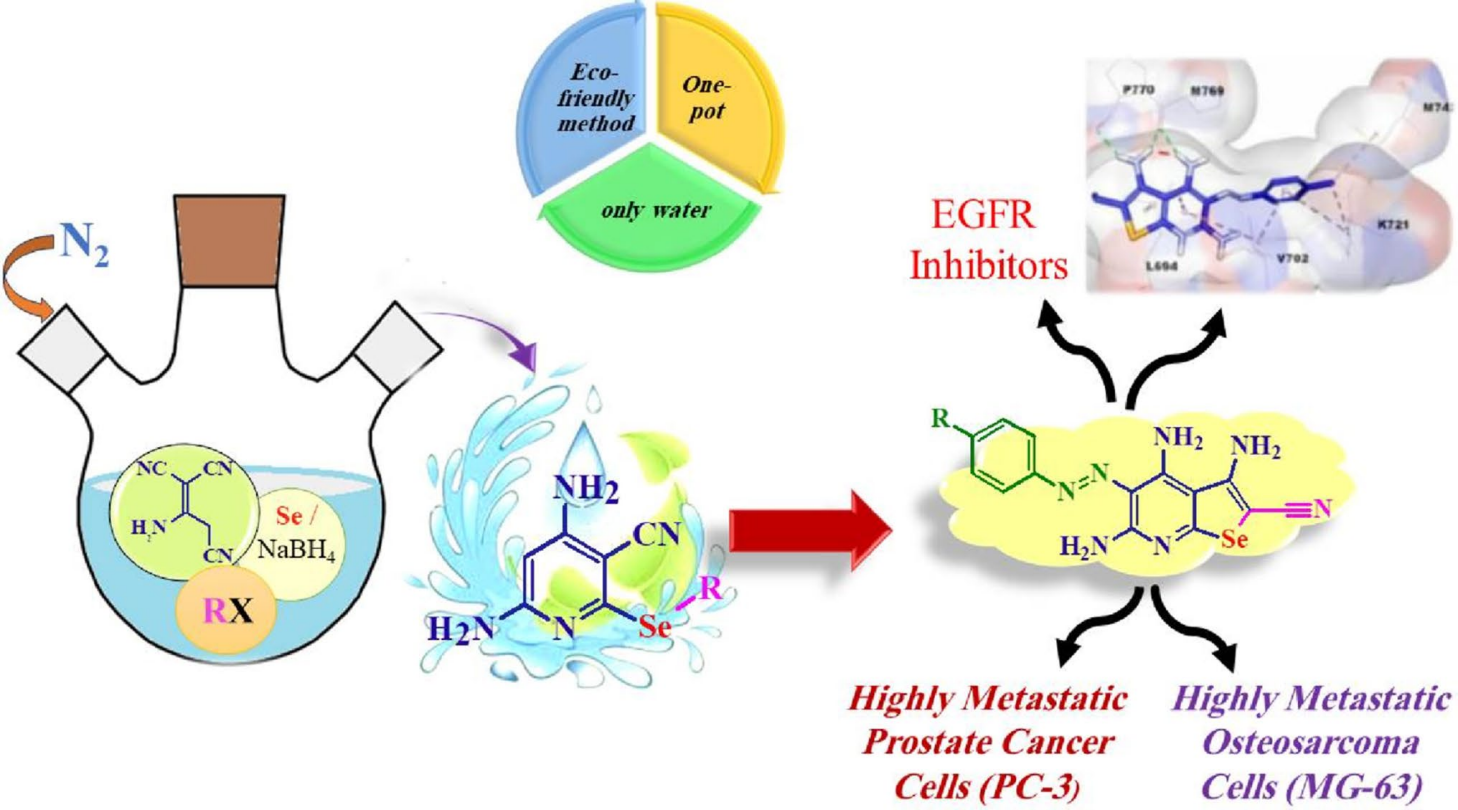

**Supplementary Information:**

The online version contains supplementary material available at 10.1007/s11030-024-10872-2.

## Introduction

Prostate cancer remains one of the most frequently diagnosed malignancies in men globally [1]. Bone metastasis is the most usual metastatic location for advanced prostate cancer, especially in the bones of the axial skeleton, such as the pelvis, spine, and ribs, where red marrow is most prevalent [2, 3]. Once bone metastasis arises, the patient survival rate drops to less than 30% [4], usually triggering several bone-related clinical manifestations such as bone pain, pathological fracture, and nerve compression syndrome that often lead to a poor prognosis for prostate cancer patients [5]. Osteosarcoma (OS) is known as the most prevalent and aggressive malignant bone disease, with a worldwide incidence of about one to three cases per million per year [6]. In addition, patients with metastatic osteosarcoma exhibit unsatisfactory responses to currently available chemotherapeutics. Therefore, discovering more effective chemotherapeutic treatments with fewer side effects is an urgent need to suppress metastasis and enhance long-term survival rates. A noteworthy concern in prostate cancer management involves identifying the factors influencing survival. Among the numerous environmental factors determining survival, particular elements have been examined, including selenium (Se) [7–9].

Selenium and Se-containing compounds have gained interest as anticancer treatments in recent decades, with numerous studies proving their great potency and selectivity against cancer cells [10, 11]. Selenium, an essential trace mineral, has been greatly researched for the development and progression of prostate cancer. These investigations have focused on its involvement in antioxidant defense [12], androgen receptor signaling [13], and cell cycle [14]. Furthermore, there is growing evidence that selenium can act as a preventive agent, and the selenium levels in the bloodstream may be correlated with the development of prostate cancer [15].

Research suggests that the epidermal growth factor receptor (EGFR), a tyrosine kinase receptor (RTK) of the ErbB family, has a substantial role in prostate cancer tumorigenesis and progression [16–18]. Previous research has linked EGFR expression to high-grade, advanced-stage prostate cancer and an increased risk of recurrence, invasion, and bone metastases [19–21]. In addition, as one of the EMT regulators, EGFR was also shown to regulate the differentiation and proliferation of osteoblasts, chondrocytes, and osteoclasts, thereby regulating cancer metastasis and bone formation [22], and thus EGFR may be considered as a surrogate marker of prostate cancer dissemination to bones [23], supporting the rationale for the use of EGFR inhibitors for prophylaxis or cure of prostate cancer metastasis [3].

Interestingly, studies tested the anticancer activity of synthetic Se-containing compounds through their suppressive EGFR, because activation of EGFR is strongly associated with tumor growth, progression, metastasis, invasion, and poor prognosis [24, 25]. As a result, there has been a boom in research into the design and synthesis of EGFR inhibitors, sparked by accumulating evidence that they hold substantial potential in cancer treatment [26, 27]. Targeting EGFR using Se-products has shown promise in treating cancer [28, 29]. Yet, there is a shortage of comprehensive investigation to evaluate the modulatory effect of Se compounds on EGFR in prostate cancer.

**Fig. 1:**
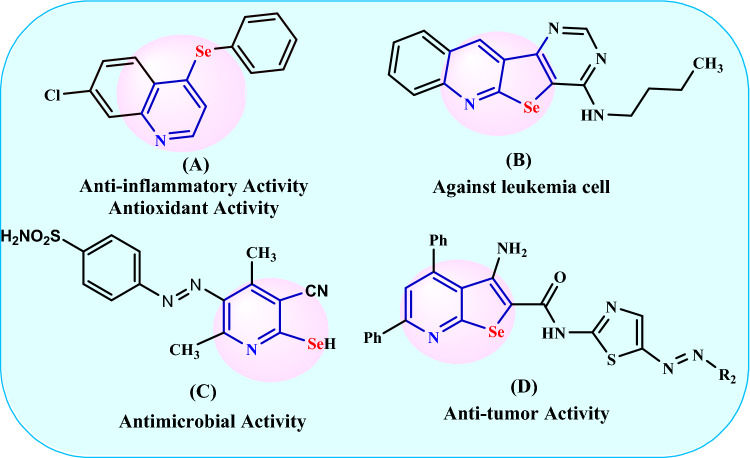
Examples of biologically active selenopyridines

Nevertheless, the synthesis of overly homogenous and stable (Se)-containing molecules remains an obstacle to their application [29]. In recent years, researchers have been more interested in organoselenium compounds and their biological effects. Selenopyridines, in particular, have received increasing attention because of their application in a variety of bioactive molecules and pharmaceuticals, such as anti-inflammatory, antioxidant activity (Fig. 1A) [30], anti-leukemic activity (Fig. 1B) [31], antimicrobial activity (Fig. 1C) [32], and antitumor activity (Fig. 1D) [33]. The anticancer characteristics of selenium and selenium-containing compounds continued to be a topic of interest in medicinal chemistry as possible scaffolds for discovering new anticancer molecules [34, 35].

**Fig. 2:**
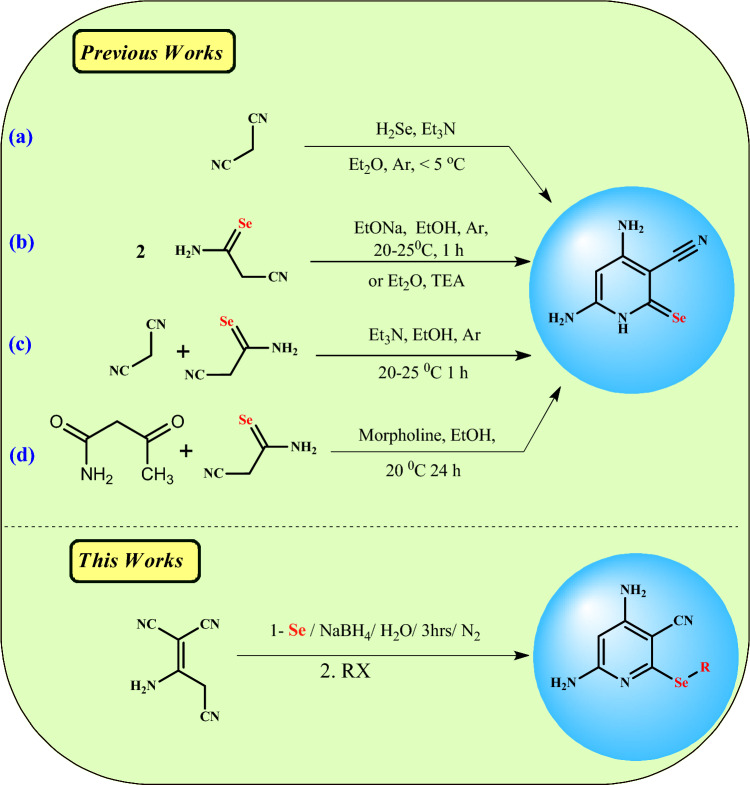
Synthetic approaches to synthesizing selenopyridines using various reaction conditions

Moreover, 4,6-Diamino-2-(alkylselanyl)pyridinonitriles were prepared by different methods: by passing H_2_Se through an alkaline solution of malononitrile (Fig. 2a) [36], alternatively, the selenopyridines can be obtained directly by the reaction of cyanoselenoacetamide [which is obtained by passing H_2_Se through a cold alkaline solution of malononitrile] with himself using EtONa as a basic catalyst [37] or by using TEA in diethyl ether [38] (Fig. 2b), as well as, selenopyridines can be obtained by the reaction of cyanoselenoacetamide with malononitrile (Fig. 2c) [38] or with 3-oxobutanamide in the basic condition (Fig. 2d) [39]. However, these methods are expensive and harmful to human health due to the use of hydrogen selenide gas, which is prepared by the reaction of selenium or selenides with acids [40] and has an unpleasant odor, is highly flammable, and is more toxic than its congener hydrogen sulfide. Furthermore, the initial effects of H_2_Se on those exposed are signs of respiratory irritation, including running noses, sneezing, irritating eyes, chest tightness, and it may be irritating to bronchitis [41, 42]. Thus, from the above mention and for the continuation of our works [43–49] we aim to use an eco-friendly method in the present work for the synthesis of selenopyridines by the utilization of NaHSe instead of toxic hydrogen selenide (Fig. 2). Moreover, we studied their reactions with different reagents optimistically to synthesize novel organoselenium compounds incorporating selenopyridine moiety and assessed the sensitivity of prostate cancer and osteosarcoma cell lines to synthesized compounds compared with Doxorubicin, standard chemotherapy using an EGFR activity inhibitory assay.

## Results and discussion

### Chemistry

**Scheme 1:**
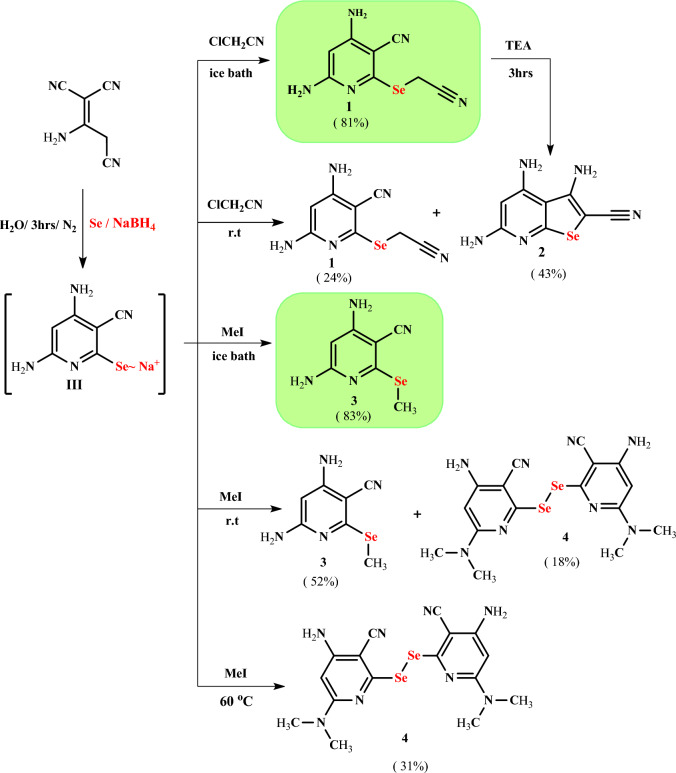
Synthesis of selenopyridine **1**–**4**

Herein, we achieved an eco-friendly method for the synthesis of novel selenopyridines via a one-pot four-component reaction of selenium, sodium borohydride, and 2-aminoprop-1-ene-1,1,3-tricarbonitrile with an active halo-compound (Scheme 1). The initial experiment was nominated in two steps. Firstly, sodium 4,6-diamin-3-cyanopyridine-2-selenolate (intermediate **III**) was synthesized by the reaction of sodium hydrogen selenide (prepared in situ by addition of sodium borohydride to a stirred solution of selenium powder in distilled water under nitrogen in an ice bath for about 10 min) with an equivalent amount of 2-aminoprop-1-ene-1,1,3-tricarbonitrile at 80 °C for 3 h under inert conditions. Secondly, the active halo-compound, such as chloroacetonitrile was added to a stirred reaction mixture at room temperature (~30 °C with continuous stirring for 1 h. This method was mentioned by TLC to afford a mixture of selenopyridine **1** (24% yield) and selenopheno[2,3-*b*]pyridine **2** (43% yield), which are separated by column chromatography (CHCl_3_:EtOH 10:1) (Table 1, entry 1).

**Table 1:** Study the effect of reaction temperature on the products percentage

Entry	RX	Temp. [°C]	Product 1 (%)	Product 2 (%)	Product 3 (%)	Product 4 (%)
1	ClCH_2_CN	30	24^a^	43^a^	–	–
2	ClCH_2_CN	40	11^a^	23^a^	–	–
3	ClCH_2_CN	50	7	13^a^	–	–
4	ClCH_2_CN	60	5^a^	10^a^	–	–
5	ClCH_2_CN	70	–^b^	–^b^	–	–
6	ClCH_2_CN	**Ice bath**	**81**^c^	0	–	–
7	MeI	30	–	–	52^a^	18^a^
8	MeI	40	–	–	36^a^	24^a^
9	MeI	50	–	–	19^a^	28^a^
10	MeI	60	–	–	0^d^	31^c^
11	MeI	70	–	–	0^d^	31^c^
12	MeI	**Ice bath**	–	–	**83**^c^	**0**^d^

^a^Isolated yield after separation and purification

^b^Other product was detected

^c^Suitable condition for compound **1**, **3** and **4**

^d^Not detected

Accordingly, the reaction was optimized and investigated to improve the yield and isolate compounds **1** or **2** as pure products by changing the reaction temperature. The effect of the temperature on the reaction was screening at various temperatures. The results of this screening indicated that by increasing the reaction temperature, the yield of selenopheno[2,3-*b*]pyridin **2** increased and the yield of selenopyridine **1** decreased, while the total yield generally decreased (Table 1, entries 2–4). The reaction didn’t occur, and no products were separated when the reaction temperature was rising to 70 °C (Table 1, entry 5). However, decreasing the reaction temperature to 0 °C leads to the formation of 4,6-diamino-2-[(cyanomethyl)seleno]pyridine-3-carbonitrile (**1**) in a good yield (81%) without any contamination by selenopheno[2,3-*b*]pyridine **2** (Table 1, entry 6). On the other hand, 3,4,6-triamino-2-cyanoselenopheno[2,3-*b*]pyridine (**2**) was formed chemically as a pure product with an excellent yield (95%) via the refluxing of selenopyridine **1** in ethanol with a catalytic amount of TEA (Scheme 1).

On the other hand, our attempt to synthesize 4,6-diamino-2-(methylselanyl)pyridine-3-carbonitrile (**3**) via the reaction of sodium 4,6-diamino-3-cyanopyridine-2-selenolate (intermediate **III**) with methyl iodide as an active halo-compound under the same condition at room temperature gave product **3** and an unexpected product 2′-diselenobis[4-amino-6-(dimethylamino)pyridine-3-carbonitrile (**4**), in low yield (Scheme 1; Table 1, entry 7). Also, methylselanylpyridine **3** (yield 19–52%) and diselenobispyridine **4** (yield 18–31%) were separated when the reaction temperature was raised from room temperature to 50 °C, which was separated by column chromatography (silica gel, eluent CHCl_3_:ethanol 10:1) (Table 1, entry 8, 9). A pure diselenobis[4-amino-6-(dimethylamino)pyridine-3-carbonitrile (**4**) was separated when the reaction temperature rose up to 60 °C (Table 1, entry 10, 11), while a pure methylselanylpyridine **3** was obtained in a good yield (83%) when the reaction occurred at 0 °C (Table 1, entry 12).

The chemical structures of the newly synthesized compounds **1**–**4** were assured by their spectral (IR, ^1^H NMR, ^13^C NMR, Dept-135) and elemental analyses (**Experimental, SI**). For example, the IR spectrum of product **4** exhibited absorption bands at 3339, 3245 cm^−1^ due to pair equivalent NH_2_, 2918 cm^−1^ due to CH aliphatic, and two equivalent nitrile groups at 2204 cm^−1^. Its ^1^H NMR spectrum showed a singlet signal corresponding to two equivalent NH_2_ at 7.13 ppm, singlet signal due to an equivalent pair of CH_pyridyl_ at 6.21 ppm, and two singlet signals at 2.42, 2.39 ppm for pair equivalent N(CH_3_)_2_ groups. Its ^13^C NMR exhibited four signals at 117.6, 95.2, 6.4, and 6.3 ppm due to the equivalent pair of all nitriles, CH_pyridyl_ and two N(CH_3_)_2_ groups, respectively. While aromatic carbons are characterized by four signals at 160.3, 159.9, 149.1 and 101.9 ppm, in addition to the XRD analysis showing the presence of selenium in the product **4**.

**Scheme 2:**
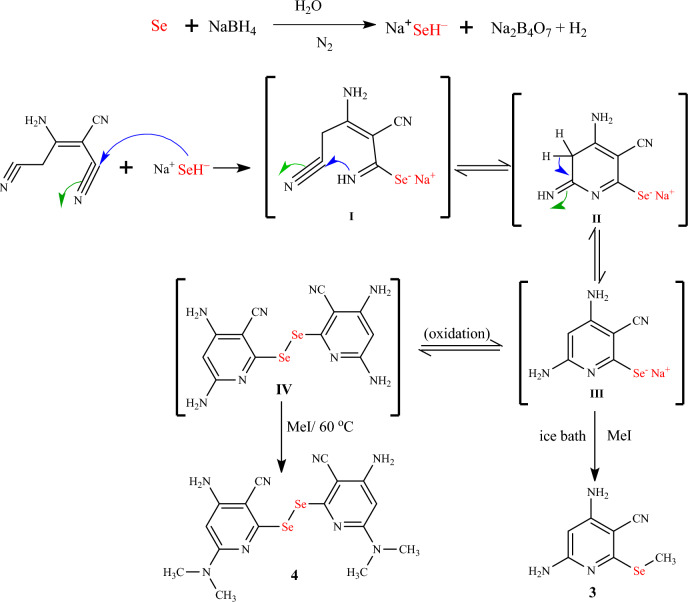
Suggestion reaction mechanism for the synthesis of selenopyridines **3** and **4**

The suggested reaction mechanism for the formation of selenopyridines **3** and **4** as shown in Scheme 2 was assumed to proceed via the reaction of 2-aminoprop-1-ene-1,1,3-tricarbonitrile (**1**) with sodium hydrogenselenide (2:1 molar ratio of borohydride to selenium in water) via nucleophilic attack of the HSe^−^ at cyano group to form sodium salt intermediate **I**. The new imino group of intermediate **I** undergoes intramolecular cyclization via the Michael addition reaction to give intermediate **II**, which rearranges to give intermediate **III**. The intermediate **III** is less stable, so it must be reacted directly with an active halo-compound (methyl iodide) at a low temperature (in an ice bath) to give *Se*-alkyl derivative **3**. If the temperature rises more than 0 °C, the intermediate **III** rapidly oxidized to give bis-selenopyridine intermediate **IV**, which reacted with methyl iodide to give *N*-alkyl derivative **4**.

**Scheme 3:**
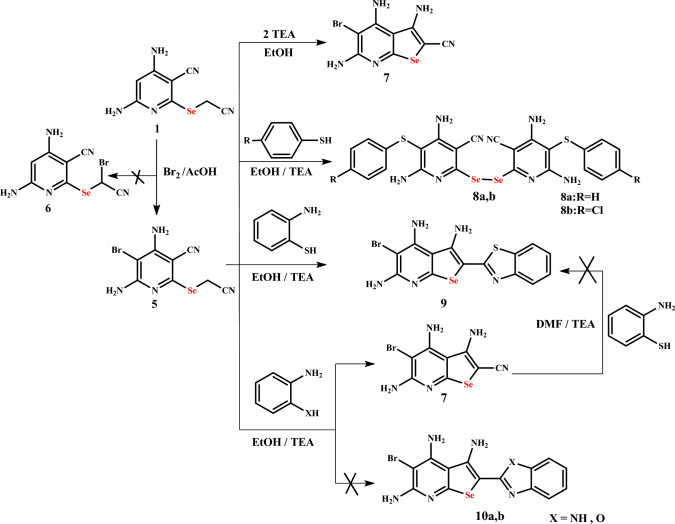
Synthesis of selenopyridine **5** and selenopheno[2,3-*b*]pyridine derivatives **7**–**9**

Also, the nucleophilic substitution reaction of selenopyridine **1** was studied via its reaction with bromine at room temperature in acetic acid to give the unexpected 4,6-diamino-5-bromo-2-[(cyanomethyl)selenyl]pyridine-3-carbonitrile (**5**) rather than 4,6-diamino-2-{[bromo(cyano)methyl]selenyl}pyridine-3-carbonitrile (**6**) (Scheme 3). The IR spectrum of bromoselenopyridine **5** showed the absence of CH aromatic and the appearance of the absorption bands corresponding to two NH_2_ at 3470, 3412, 3344, and 3232 cm^−1^; CH aliphatic at 2982 cm^−1^; two nitrile groups at 2240, 2198 cm^−1^ and C=N at 1644 cm^−1^. Moreover, its ^1^H NMR spectrum showed two singlet signals corresponding to 2NH_2_ groups at 6.92, 6.64 ppm, one singlet signal due to Se**CH**_**2**_ at 4.08 ppm, while the signal attributed to CH_pyridyl_ disappeared. Its ^13^C NMR spectrum showed two signals corresponding to two CN groups at 119.6, 116.3 ppm, one singlet signal corresponding to Se**C**H_2_ at 6.8 ppm, beside sp^2^ carbons are characterized by five signals at 157.9, 154.8, 153.6, 83.3, and 82.6 ppm.

Bromoselenopyridine **5** was used as the starting material for the synthesis of novel products, so intermolecular cyclization of **5** was carried out by refluxing it with a catalytic amount of TEA in ethanol to afford 3,4,6-triamino-5-bromoselenopheno[2,3-*b*]-pyridine-2-carbonitrile (**7**). Also, its treatment with mono-nucleophilic reagents, namely thiophenol and *p*-chlorothiophenol, in the presence of catalytic amounts of TEA afforded the unexpected 2,2′-diselanediylbis(4,6-diamino-5-(phenylthio)pyridine-3-carbonitrile) (**8a**) and 2,2′-diselanediylbis(4,6-diamino-5-(4-chlorophenylthio)pyridine-3-carbonitrile) (**8b**), respectively (Scheme 3). On the other hand, upon reacting compound **6** with aminothiophenol under the same reaction condition, 2-(benzo[*d*]thiazol-2-yl)-5-bromoselenopheno[2,3-*b*]pyridine-3,4,6-triamine (**9**) was afforded. While its reaction with *o*-phenylenediamine and/or *o*-aminophenol gave the same unexpected product **7** instead of bromoselenopheno[2,3-*b*]pyridine **10a** or **10b** (Scheme 3).

Our attempts to obtain compound **9** through the reaction of 3,4,6-triamino-5-bromoselenopheno[2,3-*b*]pyridine-2-carbonitrile (**7**) with aminothiophenol in boiling DMF in the presence of TEA as the basic catalyst failed (Scheme 3).

The IR spectrum of product **7** showed the absence of the absorption band corresponding to one C≡N group. Its ^1^H NMR spectrum showed the absence of the absorption signal corresponding to aliphatic CH_2_, while exhibiting characteristic three singlet signals corresponding to 3NH_2_ at 6.51, 6.36, and 6.15 ppm. Its ^13^C NMR spectrum showed the disappearance of two signals for one of the cyano group and aliphatic CH_2_ carbons, in addition to the increase of aromatic carbon signals at 163.7, 156.9, 154.5, 149.9, 108.0, 86.3, and 67.2 ppm, besides one signal attributed to the cyano group at 118.2 ppm. Furthermore, the IR spectrum of product **8b** showed absorption bands corresponding to pair equivalent NH_2_ groups at 3453, 3337, and 3302 cm^−1^; CH aromatic at 3085 cm^−1^, two equivalent C≡N groups at 2236 cm^−1^ and C=N at 1617 cm^−1^. Its ^1^H NMR spectrum showed the aromatic protons appeared as doublet of doublet signals at 7.52–7.45 ppm with coupling constant 19 and 8 Hz, besides two singlet signals corresponding to equivalent two NH_2_ groups at 6.59, 6.46 ppm (disappeared by D_2_O). Its ^13^C NMR spectrum showed the disappearance of signals corresponding to methylene and one of the nitrile groups, while it showed signals corresponding to equivalent two CN groups at 115.9 and nine signals for aromatic sp^2^ carbons at 159.1, 157.8, 153.7, 135.7, 133.9, 129.6, 129.4, 82.9, and 82.8 ppm. Its Dept-135 NMR spectrum shows the disappearance of the nitrile and quaternary carbon signals and exhibited two signals corresponding to phenyl carbons (two pair equivalent 2CH) at 135.7, 129.6 ppm, in addition to the XRD analysis showing the presence of selenium in the product **8b**.

Likewise, the IR spectrum of product **9** showed the disappearance of the absorption band corresponding to two C≡N groups. Its ^1^H NMR spectrum showed the evanescence of absorption signal corresponding to aliphatic CH_2_, while it exhibited three singlet signals corresponding to three NH_2_ at 6.50, 6.45, and 5.28 ppm. Whereas aromatic protons appeared as two doublet signals at 7.26 and 6.77 ppm with a coupling constant 8 Hz, in addition to two triplet signals at 7.15, 6.57 ppm with a coupling constant 7 Hz. Its ^13^C NMR spectrum showed the disappearance of three signals for two cyano groups and sp^3^ carbon of the methylene group, besides, an increase of aromatic carbon signals at 160.3, 157.6, 153.6, 151.2, 137.6, 131.5, 116.9, 116.3, 115.6, 110.7, 82.4, and 82.3 ppm.

**Scheme 4:**
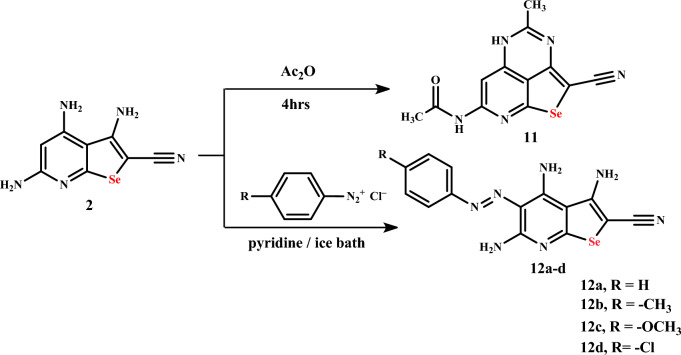
Reaction of selenopheno[2,3-*b*]pyridine **2** with Ac_2_O and ArN_2_Cl

The treatment of selenopheno[2,3-*b*]pyridine **2** with acetic anhydride afforded *N*-(2-cyano-4-methyl-5*H*-1-seleno-3,5,8-triazaacenaphthylen-7-yl)acetamide (**11**). Also, selenopheno[2,3-*b*]pyridine **2** reacted with diazonium chloride salts of aromatic amines, namely aniline, *p*-toludine, *p*-methoxyaniline, and *p*-chloroaniline, to give azo dye derivatives **12a**–**d**, respectively (Scheme 4).

The IR spectrum of seleno-3,5,8-triazaacenaphthylene **11** showed the absence of two amino groups and was exhibited by a new absorption band corresponding to 2NH groups at 3249, 3162 cm^−1^, CH_aliph._ at 2971 cm^−1^, the cyano group at 2180 cm^−1^ and the carbonyl group at 1673 cm^−1^. Its ^1^H NMR spectrum showed two new singlet signals due to two NH groups at 11.87, 10.79 ppm (disappeared by D_2_O), singlet signal at 7.62 due to CH-6, in addition to two singlet signals at 2.23 and 2.11 ppm for CH_3_ of the acetyl group and CH_3_ group, respectively. Its ^13^C NMR spectrum showed a new three signals corresponding to the carbonyl, CH_3_ acetyl group, and CH_3_ at 170.2, 24.6, and 22.4 ppm, respectively, in addition to CN and C-6 groups at 116.7 and 92.0 ppm, and the increase of aromatic carbon signals at 160.6, 158.9, 156.1, 155.5, 151.8, 145.6, and 116.2 ppm. Whereas the IR spectrum of azo dye **12a** showed absorption bands corresponding to three NH_2_ at 3399, 3369, 3309, 3212 cm^−1^; CH aromatic at 3022 cm^−1^; C≡N at 2211 cm^−1^ and C=N at 1628 cm^−1^. Its ^1^H NMR spectrum showed the disappearance of CH_pyridyl_ and the appearance of four singlet signals corresponding to three NH_2_ at 11.38, 6.97, 6.38, and 5.71 ppm, beside multiple signals for the aromatic protons at 7.43–7.05 ppm and triplet signals at 7.07 ppm due to aromatic protons with a coupling constant 7 Hz. Its ^13^C NMR spectrum showed one signal corresponding to CN groups at 115.6, while the rest of aromatic sp^2^ carbons are characterized by signals at 180.1, 160.5, 160.1, 153.6, 143.2, 129.9, 123.6, 115.0, 112.5, 111.3, and 87.4 ppm.

### Biology

#### Anticancer activity

**Fig. 3:**
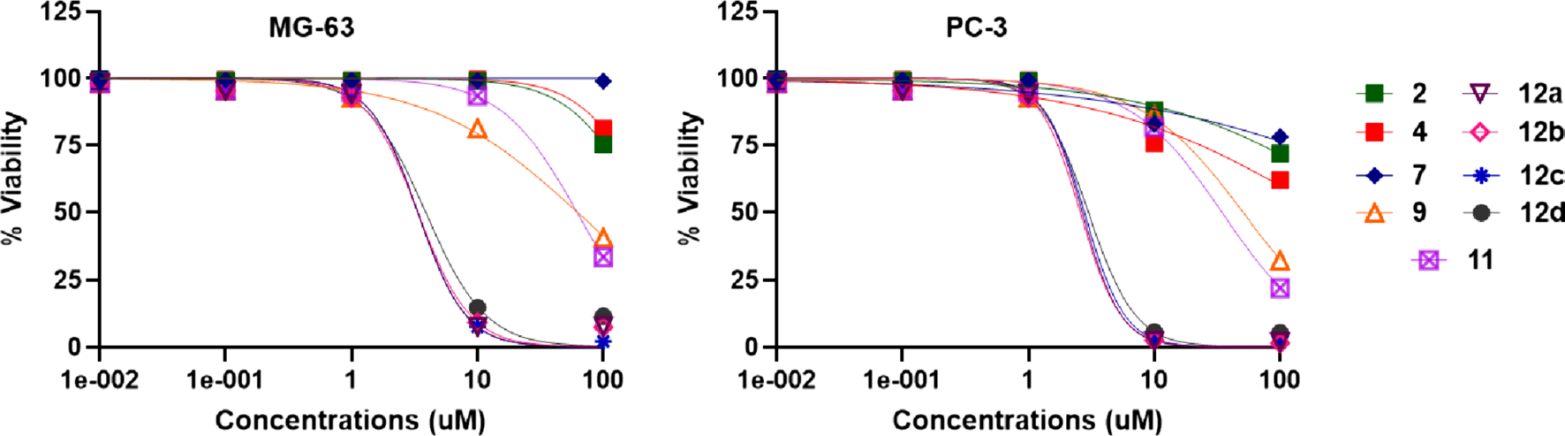
The effect of synthesized compounds against the highly metastatic cancer cell lines PC-3 and MG-63 cell lines after 72 h incubation and viability was assessed by the SRB assay. Values represent the mean ± SD of three independent experiments

Prostate cancer and osteosarcoma are incidental and relevant causes of death in humans. Hence, the discovery and characterization of innovative and effective medications is a current challenge [50]. While the early phases of prostate cancer are triggered by androgen production, the progression of tumor metastasis is usually androgen-independent [51]. Herein, we showed that PC-3 and MG-63 cells are not sensitive to **2**, **4**, and **7** without significant inhibition of cell proliferation (%) observed in both cell lines until 100 µM for each of them. As shown in Table 2 and Fig. 3, both compounds **9** and **11** exerted low cytotoxic effects (had high IC_50_ values ranging from in both cell lines), indicating lower potency being generally more potent in PC-3 than MG-63 cell line. Interestingly, we found that the compounds **12a**, **12b**, **12c**, and **12d** exhibited superior antiproliferative activities (i.e., they had IC_50_ values <4 µM) compared to doxorubicin (IC_50_ of 7.6 ± 0.45 µM in PC-3 and 9.4 ± 1.1 µM in MG-63) in both cell lines. Altogether, this experiment showed that compounds **12a**–**d** were the most potent in the panel of the investigated synthesized compounds.

**Table 2:** Anticancer activities of synthesized compounds against prostate cancer (PC-3) and osteosarcoma (MG-63) cell lines, IC_50_ values are expressed in µM as mean ± SD

Compounds	IC_50_ values (µM)	
	PC-3	MG-63
**2**	>100	>100
**4**	233.4 ± 2.6	239.5 ± 3.2
**7**	>100	>100
**9**	51.11 ± 3	62.84 ± 3.45
**11**	34.74 ± 0.77	62.17 ± 1.51
**12a**	2.69 ± 0.03	3.45 ± 0.2
**12b**	2.59 ± 0.02	3.37 ± 0.22
**12c**	2.75 ± 0.09	3.37 ± 0.12
**12d**	3.08 ± 0.01	3.93 ± 0.23
DOX	7.6 ± 0.45	9.4 ± 1.1
Lapatinib	0.13 ± 0.04	0.47 ± 0.03

#### Epidermal growth factor receptor activity inhibitory assay

EGFR-TK assay was carried out to investigate the EGFR inhibitory activity of the two most potent compounds **12a** and **12b**.

Interestingly, the results from the EGFR assay, as shown in Table 3, complement the findings of the cancer cell-based assay. Compound **12b** was the most potent anticancer agent and proved to be the highest EGFR inhibitor with IC_50_ values equal to 0.123 ± 0.004 µM which was close to the positive reference lapatinib (IC_50_ = 0.049 µM). Compound **12a** comes next with IC_50_ value equal to 0.301 µM his assay reveals that these compounds are potent EGFR inhibitors and could possibly be used as anticancer candidates.

**Table 3:** Effect of compounds **12a**, **12b** and lapatinib on EGFR

Ser	Compound	EGFR
	Code	IC_50_ (µM)
1	**12a**	0.301 ± 0.012
2	**12b**	0.123 ± 0.004
3	Lapatinib	0.049 ± 0.002

#### Docking study

**Fig. 4:**
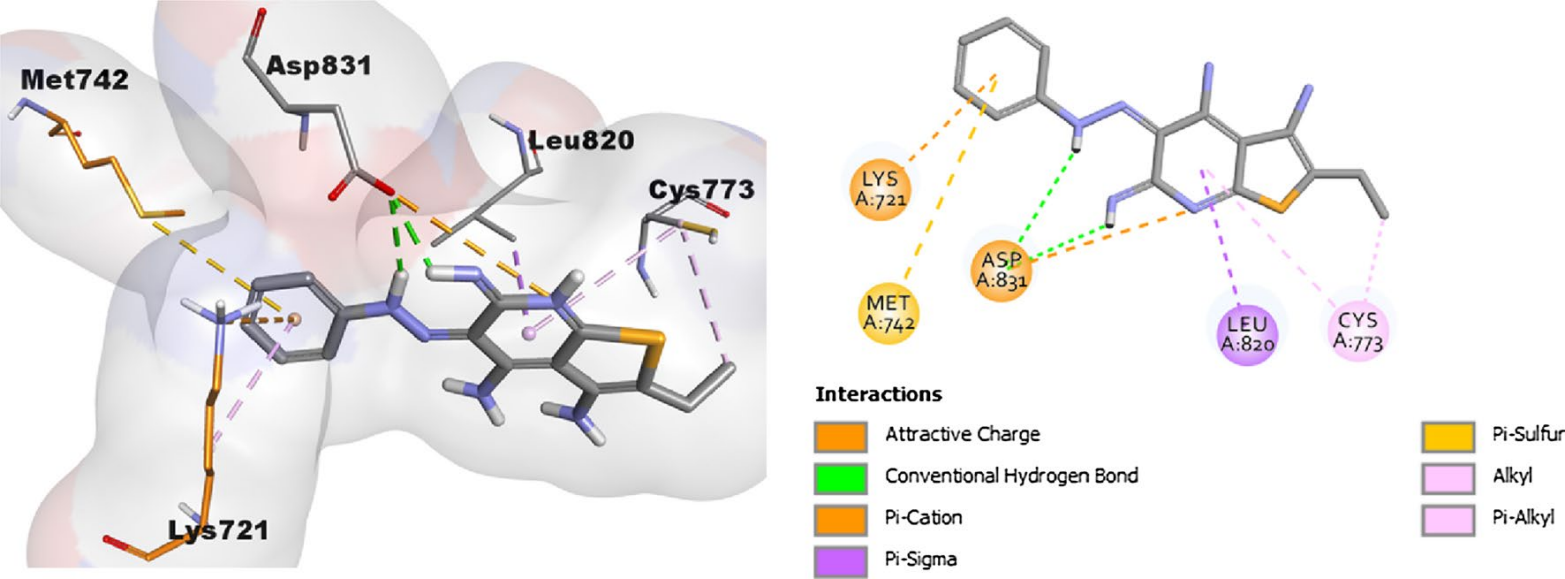
2/3D Binding modes/interactions of compound **12a** into the active site of EGFR kinase (PDB code: 1M17)

The two most potent compounds **12a** and **12b** were selected for performing the docking study to achieve some structural insights into the EGFR inhibitory activities of the newly synthesized compounds using CDOCKER embedded in the Discovery Studio software (Accelrys^®^ software corporation, San Diego, USA). The 3D crystal structure of EGFR (PDB ID: 1M17) in complex with AQ4999 was used for this docking study. As a first step, validation of the docking protocol settings was done through the re-docking of the extracted co-crystallized ligand AQ4999 from the 3D structure 1M17 using the same protocol for the docked compounds. The used docking protocol closely reproduced the bound structure with the RMSD value of 0.95 Å confirming the confidence in our docking study. Interestingly, the docking studies were consistent with the results of the EGFR inhibitory assay. The examination of docking results displayed that the two docked compounds **12a** and **12b** adopted a nearly similar disposition inside the ATP binding pocket of the EGFR. As illustrated in Fig. 4, the results of the docking study of compound **12a** (with CDOCKER interaction energy = 33.715) exhibited good fitting into the binding site of the EGFR enzyme pocket with the formation of two hydrogen bonds with Asp831 amino acids. Moreover, compound **12a** had many hydrophobic interactions such as attractive charge, pi–cation, pi–sigma, pi–sulfur, alkyl, and pi–alkyl with Asp831, Lys721, Leu820, Met742, and Cys773 amino acid residues (Fig. 4).

**Fig. 5:**
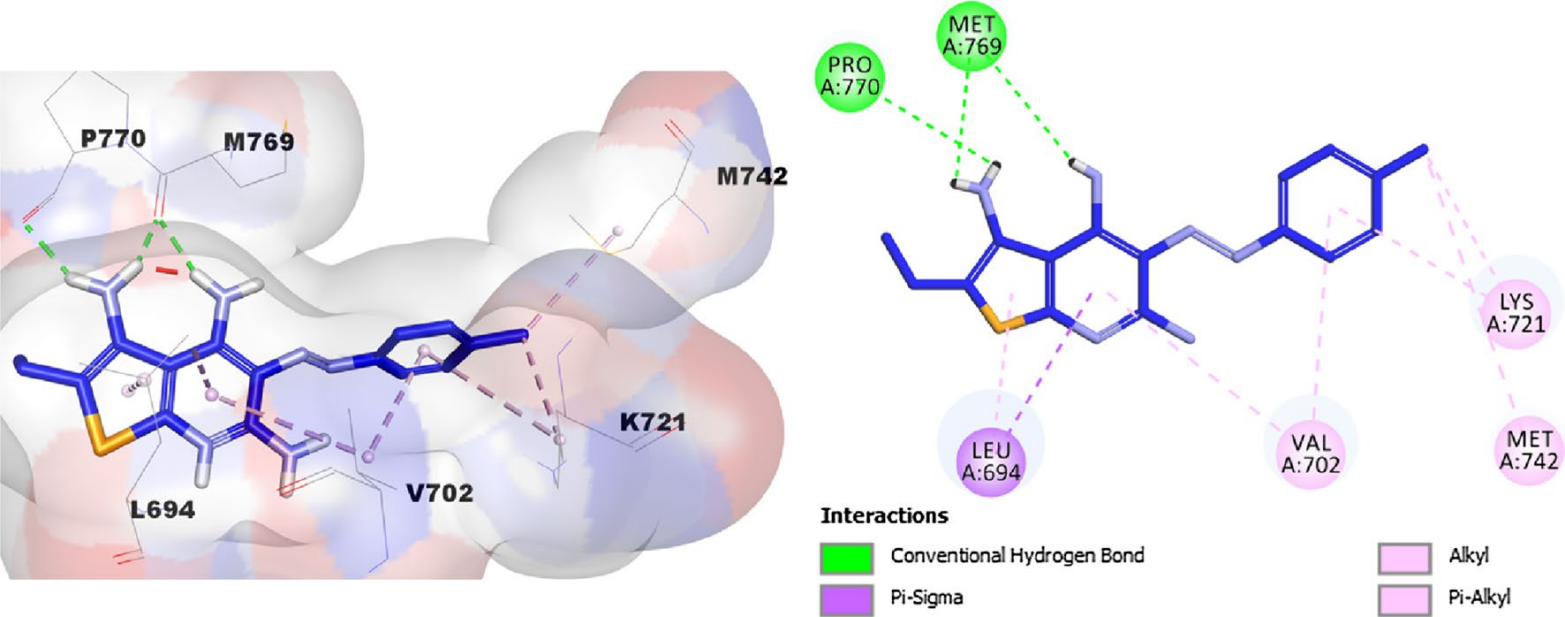
2/3D Binding modes/interactions of compound **12b** into the active site of EGFR kinase (PDB code: 1M17)

Notably, analysis of the docking results of the most potent compound **12b** (with CDOCKER interaction energy = 34.125) displayed a nice fit into the pocket of the active site of the EGFR enzyme and engaged in the formation of three hydrogen bonds with Met769 (two hydrogen bonds) and Pro770 amino acids. Additionally, compound **12b** showed many hydrophobic interactions such as Van der Waals, pi–sigma, alkyl, and pi–alkyl with Leu694, Val702, Lys721 and Met742 amino acid residues as illustrated in Fig. 5.

Altogether, the docking results are in good agreement with the anticancer activity as well as the EGFR inhibitory activity, suggesting these two compounds as promising EGFR anticancer candidates.

## Conclusion

An efficient eco-friendly method was developed for the synthesis of *Se*-alkyl selenopyridine and diselenobispyridine dervatives via a one-pot four-component reaction of selenium, sodium borohydride, 2-aminoprop-1-ene-1,1,3-tricarbonitrile and an active halo-compound through the preparation of in situ NaHSe instead of toxic hydrogen selenide. The effect of the reaction temperature was studied to prepare pure these products, which could be used as starting materials for the synthesis of novel bromoselenopyridine, selenopheno[2,3-*b*]pyridine, seleno-3,5,8-triazaacenaphthylene, and selenoazo dye derivatives. Most of the novel synthesized products have been evaluated for their in vitro anticancer activity against PC-3 and MG-63 cells. Among all, selenoazo dyes **12a**–**d** exhibited superior antiproliferative activities (i.e., they had IC_50_ values from 2.59 ± 0.02 µM to 3.93 ± 0.23 µM) compared to doxorubicin (IC_50_ of 7.6 ± 0.45 µM in PC-3 and 9.4 ± 1.1 µM in MG-63) in both cell lines. Also, the most potent compounds **12a** and **12b** proved to be potent EGFR inhibitors with IC_50_ values 0.301 and 0.123 µM, respectively, compared to lapatinib as a positive reference (IC_50_ = 0.049 µM). Moreover, the molecular docking studies for the two tested products (**12a** and **12b**) confirmed the anticancer activity and the EGFR inhibitory activity.

## Experimental

### Chemistry

#### General information

All information about reagents and spectral analyses were showed in Supporting Information.

#### General procedure for synthesis of selenopyridine derivatives 1 and 3

2-Aminoprop-1-ene-1,1,3-tricarbonitrile (1 g, 7.5 mmol) in 50 mL of ethanol was added to a solution of sodium hydrogen selenide [freshly prepared from finely divided selenium powder (0.59 g, 7.5 mmol) and sodium borohydride (0.56 g, 15 mmol) in 20 mL water] and the reaction mixture was refluxed under nitrogen conditions for 3 h., then cooled to room temperature and the active halo-compounds such as: chloroacetonitrile (0.57 g, 7.5 mmol) and/or methyl iodide (1 g, 7.5 mmol) was added dropwise with stirring in an ice bath for 1 h. After completion of the reaction (monitored by TLC, eluent CHCl_3_:ethanol 10:1, Rf_**1**_ = 0.37, and Rf_**3**_ = 0.41), the formed precipitate was collected by filtration, washed several times with water, dried, and recrystallized from ethanol.

##### 4,6-Diamino-2-[(cyanomethyl)selenopyridine-3-carbonitrile (1)

White crystals, yield 1.54 g (81%); mp. 160–162 °C, Lit. mp. 158–160 ℃ [38]; FT-IR (ATR) *ν*_*max*_: 3357, 3316, 3240 (2NH_2_), 3032 (CH_arom._), 2974 (CH_aliph._), 2234, 2190 (2C≡N), 1637 (C=N) cm^−1^; ^1^H NMR (400 MHz, DMSO-*d*_*6*_): *δ* 6.48 (s, 2H, NH_2_ exchanged by D_2_O), 6.39 (s, 2H, NH_2_ exchanged by D_2_O), 5.53 (s, 1H, CH_pyridyl_), 4.06 (s, 2H, CH_2_) ppm; ^13^C NMR (100 MHz, DMSO-*d*_*6*_): *δ* 161.2, 157.3, 155.9, 119.8 (CN), 117.2 (CN), 85.9 (CH_pyridyl_), 82.7, 6.1 (CH_2_) ppm. Dept-135 NMR (100 MHz, DMSO-*d*_*6*_): *δ* 86.1 (CH_pyridyl_), 6.1 (CH_2_, exchangeable) ppm. *Anal*. Calcd. For C_8_H_7_N_5_Se (252.13): C, 38.11; H, 2.80; N, 27.78% Found: C, 38.28; H, 2.93; N, 27.63%.

#### 4,6-Diamino-2-(methylselanyl)pyridine-3-carbonitrile (3)

White crystals, yield 1.43 g (83%); mp. 148–150 °C, Lit. mp. 150–152 ℃ [38]; FT-IR (ATR)* ν*_*max*_: 3424, 3352, 3329, 3246 (2NH_2_), 2933 (CH_aliph._), 2203 (C≡N), 1623 (C=N) cm^−1^; ^1^H NMR (400 MHz, DMSO-*d*_*6*_): *δ* 6.30 (s, 2H, NH_2_), 6.19 (s, 2H, NH_2_), 5.47 (s, 1H, CH_pyridyl_), 2.35 (SeCH_3_) ppm; ^13^C NMR (100 MHz, DMSO-*d*_*6*_): *δ* 161.0, 158.9, 157.1, 117.9 (CN), 85.5 (CH_pyridyl_), 83.3, 5.7 (SeCH_3_) ppm. *Anal*. Calcd. For C_7_H_8_N_4_Se (227.12): C, 37.02; H, 3.55; N, 24.67%. Found: C, 37.16; H, 3.38; N, 24.81%.

#### Synthesis of 3,4,6-triamino-2-cyanoselenopheno[2,3-*b*]pyridine (2)

A mixture of selenopyridine **1** (1 g, 4 mmol) and TEA (0.4 g, 4 mmol) in 20 mL of ethanol was refluxed for 3 h. After completion of the reaction (monitored using TLC, eluent CHCl_3_:ethanol 10:1, Rf = 0.18), the excess solvent was evaporated under vacuum. The resulting product was collected and recrystallized from ethanol. Brown crystals yield 0.48 g (95%); mp. 258–260 °C, Lit mp. 256–258 ℃ [38]; FT-IR (ATR)* ν*_*max*_: 3416, 3315, 3269, 3201 (3NH_2_), 2164 (C≡N), 1661 (C=N) cm^−1^; ^1^H NMR (400 MHz, DMSO-*d*_*6*_): *δ* 6.16 (s, 4H, 2NH_2_), 6.11 (s, 2H, NH_2_), 5.64 (s, 1H, CH) ppm; ^13^C NMR (100 MHz, DMSO-*d*_*6*_): *δ* 166.0, 161.0, 155.3, 153.8, 118.9 (CN), 107.5, 88.9 (CH), 62.8 ppm. *Anal*. Calcd. For C_8_H_7_N_5_Se (252.13): C, 38.11; H, 2.80; N, 27.78%. Found: C, 37.97; H, 2.92; N, 27.81%.

#### Synthesis of 2,2′-diselenobis[4-amino-6-(dimethylamino)pyridine-3-carbonitrile (4)

2-Aminoprop-1-ene-1,1,3-tricarbonitrile (1 g, 7.5 mmol) in 50 mL of ethanol was added to a solution of sodium hydrogen selenide [freshly prepared from finely divided selenium powder (0.59 g, 7.5 mmol) and sodium borohydride (0.56 g, 15 mmol) in 20 mL of water] and the reaction mixture was refluxed under nitrogen conditions for 3 h, then cooled to 60 ℃, and methyl iodide (1 g, 7.5 mmol) was added dropwise with stirring for 1 h at 60 °C. After completion of the reaction (monitored by TLC, eluent CHCl_3_:ethanol 10:1, Rf = 0.74), the formed precipitate was collected, washed with water, dried and recrystallized from ethanol. Yellow powder, yield 1.1 g (31%); mp. 172–174 °C; FT-IR (ATR)* ν*_*max*_: 3339, 3245 (NH_2_), 3043 (CH_arom._), 2918 (CH_aliph._), 2204 (C≡N), 1628 (C=N) cm^−1^; ^1^H NMR (400 MHz, DMSO-*d*_*6*_): *δ* 7.13 (s, 4H, 2NH_2_), 6.21 (s, 2H, 2CH_pyridyl_), 2.42 (s, 6H, N(CH_3_)_2_), 2.39 (s, 6H, N(CH_3_)_2_) ppm; ^13^C NMR (100 MHz, DMSO-*d*_*6*_): *δ* 160.3, 159.9, 149.1, 117.6 (CN), 101.9, 95.2 (CH_pyridyl_), 6.4 (2NCH_3_), 6.3 (2NCH_3_) ppm. *Anal*. Calcd. For C_16_H_18_N_8_Se_2_ (480.28): C, 40.01; H, 3.78; N, 23.33%. Found: C, 40.23; H, 3.62; N, 23.29%.

#### Synthesis of 4,6-diamino-5-bromo-2-(cyanomethylselanyl)nicotinonitrile (5)

To a solution of compound **1** (0.25 g, 2 mmol) in acetic acid (20 mL), bromine solution (0.31 g, 2 mmol) in acetic acid (5 mL) was added dropwise with stirring for about 30 min at room temperature in the presence of sunlight. After completion of the reaction (monitored by TLC, eluent CHCl_3_:ethanol 10:1, Rf = 0.55), the formed precipitate was collected by filtration, washed by distilled water several times, dried, and recrystallized from ethanol. Brown crystal, yield 0.54 g (82%); mp. 176–178 °C; FT-IR (ATR) *ν*_*max*_: 3470, 3412, 3344, 3232 (2NH_2_), 2982 (CH_aliph._), 2240, 2198 (2C≡N), 1644 (C=N) cm^−1^; ^1^H NMR (400 MHz, DMSO-*d*_*6*_): *δ* 6.92 (s, 2H, NH_2_), 6.64 (s, H, NH_2_), 4.08 (s, 2H, SeCH_2_) ppm; ^13^C NMR (100 MHz, DMSO-*d*_*6*_): *δ* 157.9, 154.8, 153.6, 119.6 (**C**N), 116.3 (**C**N), 83.3, 82.6, 6.8 (Se**C**H_2_) ppm. *Anal*. Calcd. For C_8_H_6_BrN_5_Se (331.03): C, 29.03; H, 1.83; N, 21.16%. Found: C, 29.21; H, 1.73; N, 21.32%.

#### Synthesis of 3,4,6-triamino-5-bromoselenopheno[2,3-*b*]pyridine-2-carbonitrile (7)

A mixture of selenopyridine **5** (1 g, 3 mmol) and TEA (0.3 g, 3 mmol) in 20 mL of ethanol was refluxed for 3 h. After completion of the reaction (monitored by TLC, silica gel, eluent CHCl_3_:ethanol 10:1, Rf = 0.29), the formed precipitate was filtrated and recrystallized from acetone. Brown crystals, yield 0.86 g (86%); mp. 268–270 °C; FT-IR (ATR) *ν*_*max*_: 3420, 3333, 3269 (3NH_2_), 3051 (CH_arom._) 2177 (C≡N) cm^−1^; ^1^H NMR (400 MHz, DMSO-*d*_*6*_): *δ* 6.51 (s, 2H, NH_2_), 6.36 (s, 2H, NH_2_), 6.15 (s, 2H, NH_2_) ppm; ^13^C NMR (100 MHz, DMSO-*d*_*6*_): *δ* 163.7, 156.9, 154.5, 149.9, 118.2 (CN), 108.0, 86.3, 67.2 ppm. *Anal*. Calcd. For C_8_H_6_BrN_5_Se (331.03): C, 29.03; H, 1.83; N, 21.16%. Found: C, 29.12; H, 1.72; N, 21.21%.

#### General procedure for synthesis of compounds 8a,b

A mixture of compound **5** (0.5 g, 1.5 mmol), and (1.5 mmol) an appropriate thiol namely: thiophenol (0.17 g, 1.5 mmol (**Method A**) and/or 0.34 g, 3 mmol (**Method B**) and/or *p*-chlorothiophenol (0.22 g, 1.5 mmol (**Method A**) and/or 0.44 g, 3 mmol (**Method B**) with a catalytic amount of TEA dissolved in ethanol (30 mL) and refluxed for 8 h. After completion of the reaction (monitored by TLC, eluent CHCl_3_:ethanol 10:1, Rf_**8a**_ = 0.70, Rf_**8b**_ = 0.74), the excess solvent was evaporated under vacuum. The resulting product was collected and recrystallized from the appropriate solvent.

#### 2,2'-Diselanediylbis(4,6-diamino-5-(phenylthio)pyridine-3-carbonitrile) (8a)

Brown crystal (ethanol); yield (30% Method** A**, 62% Method** B**); mp. 198–200 °C; FT-IR (ATR) *ν*_*max*_: 3450, 3335, 3304 (2NH_2_), 3079 (CH_arom._), 2236 (C≡N), 1617 (C=N) cm^−1^; ^1^H NMR (400 MHz, DMSO-*d*_*6*_): *δ* 7.50–7.47 (m, 4H, CH_arom._), 7.43–7.39 (m, 6H, CH_arom._), 6.62 (s, 4H, 2NH_2_), 6.51 (s, 4H, 2NH_2_) ppm; ^13^C NMR (100 MHz, DMSO-*d*_*6*_): *δ* 159.5, 157.8, 153.7, 133.8, 130.6, 129.7, 128.9, 116.1 (CN), 83.3, 82.9 ppm. *Anal*. Calcd. For C_24_H_18_N_8_S_2_Se_2_ (640.50): C, 45.00; H, 2.83; N, 17.49%. Found: C, 45.11; H, 2.91; N, 17.40%.

#### 2,2'-Diselanediylbis(4,6-diamino-5-(4-chlorophenylthio)pyridine-3-carbonitrile) (8b)

Yellow crystal (acetone); yield (33% Method **A**, 70% Method **B**); mp. 236–238 °C; FT-IR (ATR) *ν*_*max*_: 3453, 3337, 3302 (2NH_2_), 3085 (CH_arom._), 2236 (C≡N), 1617 (C=N) cm^−1^; ^1^H NMR (400 MHz, DMSO-*d*_*6*_): *δ* 7.52–7.45 (dd, 8H, *J* = 19, 8 Hz, CH_arom._), 6.59 (s, 4H, 2NH_2_ exchanged by D_2_O), 6.46 (s, 4H, 2NH_2_ exchanged by D_2_O) ppm; ^13^C NMR (100 MHz, DMSO-*d*_*6*_): *δ* 159.1, 157.8, 153.7, 135.7, 133.9, 129.6, 129.4, 115.9 (CN), 82.9, 82.8 ppm; Dept-135 NMR (100 MHz, DMSO-*d*_*6*_): *δ* 135.7 (CH_arom_), 129.6 (CH_arom_) ppm. *Anal*. Calcd. For C_24_H_16_C_l2_N_8_S_2_Se_2_: (711.39): C, 40.63; H, 2.27; N, 15.80%. Found: C, 40.70; H, 2.19; N, 15.68%.

#### Synthesis of 2-(benzo[*d*]thiazol-2-yl)-5-bromo-selenopheno[2,3-*b*]-pyridine-3,4,6-triamine (9)

A mixture of compound **5** (1 g, 3 mmol) and *o*-aminothiophenol (0.377 g, 3 mmol) was refluxed in ethanol (30 mL) for 6 h in the presence of catalytic amount of TEA. After completion of the reaction (monitored by TLC, eluent CHCl_3_:ethanol 10:1, Rf = 0.59), the formed precipitate was filtrated and recrystallized from acetone. Yellow crystal, yield 0.45 g (68%); mp. dec. 270–272 °C; FT-IR (ATR) *ν*_*max*_: 3463, 3426, 3348 (3NH_2_), 3071 (CH_arom._), 1623 (C=N) cm^−1^; ^1^H NMR (400 MHz, DMSO-*d*_*6*_): *δ* 7.26 (d, 1H, *J* = 8 Hz, CH_arom._), 7.15 (t, 1H, *J* = 7 Hz, CH_arom._), 6.77 (d, 1H, *J* = 8 Hz, CH_arom._), 6.57 (t, 1H, *J* = 7 Hz, CH_arom._), 6.50 (s, 2H, NH_2_), 6.45 (s, 2H, NH_2_), 5.28 (s, 2H, NH_2_) ppm; ^13^C NMR (100 MHz, DMSO-*d*_*6*_): *δ* 160.3, 157.6, 153.6, 151.2, 137.6, 131.5, 116.9, 116.3, 115.6, 110.7, 82.4, 82.3 ppm. *Anal*. Calcd. For C_14_H_10_BrN_5_SSe (439.19): C, 38.29; H, 2.29; N, 15.95% Found: C, 38.40; H, 2.19; N, 15.89%.

#### Synthesis of *N*-(2-cyano-4-methyl-5*H*-1-seleno-3,5,8-triazaacenaphthylen-7-yl)acetamide (11)

Selenopheno[2,3-*b*]pyridine **1** (0.5 g, 2 mmol) was refluxed in 15 mL of acetic anhydride for 3 h and allowed to cool at room temperature, then poured into 50 mL of cold water and left to stand for 1 h. After completion of the reaction (monitored by TLC, eluent CHCl_3_:ethanol 10:1, Rf = 0.25), the formed precipitate was filtered off, washed with distilled water several times, dried, and recrystallized from ethanol. White powder, yield 0.49 g (78%); mp. ˃300 °C; FT-IR (ATR) *ν*_*max*_: 3249, 3162, (2NH), 3013 (CH_arom._), 2971 (CH_aliph._), 2180 (C≡N), 1673 (C=O) cm^−1^; ^1^H NMR (400 MHz, DMSO-*d*_*6*_): *δ* 11.87 (s, 1H, NH exchanged by D_2_O), 10.79 (s, 1H, NH exchanged by D_2_O), 7.62 (s, 1H, CH-6_._), 2.23 (s, 3H, CO**CH**_**3**_), 2.11 (s, 3H, CH_3_) ppm; ^13^C NMR (100 MHz, DMSO-*d*_*6*_): *δ* 170.2 (C=O), 160.6, 158.9, 156.1, 155.5, 151.8, 145.6, 116.7 (CN), 116.2, 92.0, 24.6 (CO**CH**_**3**_), 22.4 (CH_3_) ppm. *Anal*. Calcd. For C_12_H_9_N_5_OSe (318.19): C, 45.30; H, 2.85; N, 22.01% Found: C, 45.39; H, 2.74; N, 22.09%.

#### General procedure for synthesis of 3,4,6-triamino-5-[aryldiazenyl]selenopheno[2,3-*b*]pyridine-2-carbonitrile 12a–d

Sodium nitrite (0.14 g, 2 mmol) in 2 mL of cold water was added slowly at 0–5 °C to a stirred solution of appropriate aromatic amines (2 mmol) namely aniline (0.18 g), *p*-toluidine (0.21 g), *p*-methoxyaniline (0.24 g) and *p*-chloroaniline (0.25 g) in 5 mL of conc. HCl. The formed diazonium salt solution was added with continuous stirring to an ice cooled solution of selenopheno[2,3-*b*]pyridine **2** (0.5 g, 2 mmol) in 20 mL of pyridine at 0–5 °C. The reaction mixture was allowed to stand for 30 min (in an ice bath) and then poured into 50 mL cold water. After completion of the reaction (monitored by TLC, eluent CHCl_3_:ethanol 10:1, **12a**–**d**, Rf = 0.40, 0.48, 0.37, 0.40, respectively), the formed precipitate filtered off, washed with distilled water several times, dried, and recrystallized from dioxane.

#### 3,4,6-Triamino-5-[phenyldiazenyl]selenopheno[2,3-*b*]pyridine-2-carbonitrile (12a)

Beige powder, yield 0.6 g (85%); mp. dec 240–242 °C; FT-IR (ATR) *ν*_*max*_: 3399, 3369, 3309, 3212 (3NH_2_), 3022 (CH_arom._), 2211 (C≡N), 1628 (C=N) cm^−1^; ^1^H NMR (400 MHz, DMSO-*d*_*6*_): *δ* 11.38 (s, 1H, NH), 7.43–7.35 (m, 4H, CH_arom._), 7.07 (t, 1H, *J* = 7 Hz, CH_arom._) 6.97 (s, 2H, NH_2_), 6.38 (s, 2H, NH_2_), 5.71 (s, 1H, NH) ppm.^13^C NMR (100 MHz, DMSO-*d*_*6*_): *δ* 180.1, 160.5, 160.1, 153.6, 143.2, 129.9, 123.6, 115.6 (CN), 115.0, 112.5, 111.3, 87.4 ppm. UV–Vis (λ_max_, nm, DMSO): 378 nm. *Anal*. Calcd. For C_14_H_11_N_7_Se (356.24): C, 47.20; H, 3.11; N, 27.52%. Found: C, 47.36; H, 3.32; N, 27.74%.

#### 3,4,6-Triamino-5-(4-methylphenyl)diazenylselenopheno[2,3-*b*]pyridine-2-carbonitrile (12b)

Yellow powder, yield 0.65 g (89%); mp. dec. 220–222 °C; FT-IR (ATR) *ν*_*max*_: 3383, 3300, 3173 (3NH_2_), 3017 (CH_arom._), 2962 (CH_aliph._), 2213 (C≡N), 1647 (C=N) cm^−1^; ^1^H NMR (400 MHz, DMSO-*d*_*6*_): *δ* 11.32 (s, 1H, NH), 7.30 (d, 2H, *J* = 8 Hz, CH_arom._), 7.17 (d, 2H, *J* = 8 Hz, CH_arom._), 6.98 (s, 2H, NH_2_), 6.35 (s, 2H, NH_2_), 5.69 (s, 1H, NH), 2.27 (s, 3H, CH_3_) ppm; ^13^C NMR (100 MHz, DMSO-*d*_*6*_): *δ* 180.1, 160.5, 160.2, 153.6, 140.9, 132.8, 130.3, 115.7 (CN), 114.4, 112.6, 111.4, 87.4, 20.8 (CH_3_) ppm; UV–Vis (λ_max_, nm, DMSO): 380 nm. *Anal*. Calcd. For C_15_H_13_N_7_Se (370.27): C, 48.66; H, 3.54; N, 26.48%. Found: C, 48.51; H, 3.73; N, 26.65%.

#### 3,4,6-Triamino-5-[(4-methoxyphenyl)diazenyl]selenopheno[2,3-*b*]pyridine-2-carbonitrile (12c)

Reddish brown powder, yield 0.66 g (87%); mp. dec. 278–280 °C; FT-IR (ATR) *ν*_*max*_: 3399, 3302, 3173 (3NH_2_), 3042 (CH_arom._), 2836 (CH_aliph._), 2210 (C≡N), 1658 (C=N) cm^−1^; ^1^H NMR (400 MHz, DMSO-*d*_*6*_): *δ* 11.37 (s, 1H, NH), 7.53 (s, 2H, NH_2_), 7.34 (d, 2H, *J* = 8 Hz, CH_arom._), 7.02 (s, 2H, NH_2_), 6.96 (d, 2H, *J* = 8 Hz, CH_arom._), 5.74 (s, 1H, NH), 3.75 (s, 3H, OCH_3_) ppm; ^13^C NMR (100 MHz, DMSO-*d*_*6*_): *δ* 175.8, 159.7, 157.9, 156.3, 154.3, 136.6, 117.3 (CN), 115.2, 113.4, 112.6, 112.1, 87.1, 55.8 (OCH_3_) ppm; UV–Vis (λ_max_, nm, DMSO): 382 nm. *Anal*. Calcd. For C_15_H_13_N_7_OSe (386.27): C, 46.64; H, 3.39; N, 25.38%. Found: C, 46.72; H, 3.28; N, 25.47%.

#### 3,4,6-Triamino-5-[(4-chlorophenyl)diazenyl]selenopheno[2,3-*b*]pyridine-2-carbonitrile (12d)

Dark yellow powder, yield 0.7 g (92%); mp. dec. 246–248 °C; FT-IR (ATR) *ν*_*max*_: 3323, 3242, 3202 (3NH_2_), 3060 (CH_arom._), 2215 (C≡N), 1616 (C=N) cm^−1^; ^1^H NMR (400 MHz, DMSO-*d*_*6*_): *δ* 11.46 (br, 1H, NH), 7.41 (s, 4H, CH_arom._), 6.81 (s, 2H, NH_2_), 6.36 (s, 2H, NH_2_), 5.68 (s, 1H, NH) ppm; ^13^C NMR (100 MHz, DMSO-*d*_*6*_): *δ* 179.8, 160.4, 159.9, 153.5, 142.2, 129.7, 127.2, 117.2 (CN), 115.3, 112.4, 111.3, 87.5 ppm; UV–Vis (λ_max_, nm, DMSO): 378 nm. *Anal*. Calcd. For C_14_H_10_ClN_7_Se (390.68): C, 43.04; H, 2.58; N, 25.10%. Found: C, 43.19; H, 2.42; N, 25.27%.

### Biology

#### Anticancer activity

##### Cell lines and culture conditions

All details about PC3 and MG-63 cells, in addition to the culture conditions were explained in Supporting Information.

##### Assessment of cytotoxicity by SRB assay

The SRB assay was used to evaluate the effect of the synthesized selenium containing heterocyclic compounds on cancer cells according to the previous literatures [52, 53] (see Supporting Information).

##### EGFR inhibitory assay

A cell-free assay was used to investigate the mechanism of inhibition of EGFR kinase according to the reported method [54] (see Supporting Information).

##### Statistical analysis

All details about statistical analysis were showed in Supporting Information.

#### Docking study

The docking study was accomplished by using Discovery Studio software 2016 client v16.1.0.15350 (San Diego, CA) with the CDOCKER program and the 3.5 Å 3D structure of EGFR (PDB ID: 1M17) [54] in complex with AQ4999 was downloaded from the protein data bank (see Supporting Information).

## Supplementary Information

Below is the link to the electronic supplementary material.

Supplementary file1 (DOCX 2940 KB)
