# Supplementary material for: An efficient methodological approach for synthesis of selenopyridines: generation, reactions, anticancer activity, EGFR inhibitory activity and molecular docking studies
Source: Mol Divers. 2024 May 13;29(1):519–34. doi: 10.1007/s11030-024-10872-2 (PMC11785687; doi:10.1007/s11030-024-10872-2)

**Supporting Information**

**An efficient methodological approach for synthesis of selenopyridines: generation, reactions, anticancer activity, EGFR inhibitory activity and molecular docking studies**

Bahgat R. M. Hussein^1,^*, Sham M. M. El-Saghier^1^, Rasha M. Allam^2^, Mamdouh F. A. Mohamed^3^, Amer A. Amer^1,^*

*^1^ Department of Chemistry, Faculty of Science, Sohag University, Sohag, 82524, Egypt.*

*^2^ Pharmacology Department, National Research Centre, Giza 11865, Egypt.*

*^3^Department of Pharmaceutical Chemistry, Faculty of Pharmacy, Sohag University, 82524 Sohag, Egypt.*

***E-mails**: [bahgat.ramadan@yahoo.com](mailto:bahgat.ramadan@yahoo.com) & [bahgat@science.sohag.edu.eg](mailto:bahgat@science.sohag.edu.eg) (B.R.M. Hussein), [amer_chem@yahoo.com](mailto:amer_chem@yahoo.com) (A.A. Amer).

**Mobil:** +201064382209 (B.R.M. Hussein), +201117860798 (A.A. Amer).

1. **Chemistry:**

# General Information:

All reactions were monitored by thin layer chromatography (TLC) using precoated plates of silica gel G/ UV-254 of 0.25 mm thickness (Merck 60F254) using UV light (254 nm/365 nm) for visualization All melting points were recorded by Kofeler melting point apparatus and uncorrected. IR spectra were measured as KBr pellets on a spectrometer FT-IR spectrophotometer. ^1^H NMR , ^13^C NMR and Dept-135 (DMSO-*d_6_*) spectra were recorded at 400 MHz and 100 MHz, respectively on Bruker Bio Spin AG at Sohag University, For ^1^H NMR, chemical shifts (δ) were given in parts per million (ppm) with reference to tetramethylsilane (TMS) as an internal standard (δ = 0 ppm); coupling constants (J) were given in hertz (Hz) and data are reported as follows: chemical shift, integration, multiplicity (s = singlet, d = doublet, t = triplet, m = multiplet, dd= doublet of doublets). For ^13^C NMR, TMS (δ = 0 ppm) or DMSO (δ = 39.51 ppm) was used as internal standard. Elemental analyses were obtained on a Perkin-Elmer CHN analyzer model. Ultraviolet-Visible (UV-Vis) absorption spectra were recorded on T80 UV-Vis spectrophotometer. XRD data of the LaFeO_3_ NPs were collected with a D8 Advance with DAVINCI design (Bruker, Germany) ), using as X-ray source the Cu Kα radiation (wavelength λ = 1.5418 Å), at 40 kV and 40 mA, a 2θ range of 5–90°, a step size of 0.02°, and a time/step of 0.6 s. A Si zero-background sample holder was used, operated by DIFFRAC. Measurements Center Version V7.3.0 (32Bit) software, while the assignment of peaks was based on the Powder Diffraction Files (PDF) of the COD database (Crystallography Open Database).

- 1. **General procedure for synthesis of selenopyridine derivatives 1 and 3:**

2-Aminoprop-1-ene-1,1,3-tricarbonitrile (1 g, 7.5 mmol ) in 50 mL of ethanol was added to a solution of sodium hydrogen selenide [freshly prepared from finely divided selenium powder (0.59 g, 7.5 mmol) and sodium borohydride (0.56 g, 15 mmol) in 20 mL water] and the reaction mixture was refluxed under nitrogen conditions for 3 hrs., then cooled to room temperature and the active halo-compounds such as: chloroacetonitrile (0.57 g, 7.5 mmol) and/or methyl iodide (1 g, 7.5 mmol) was added dropwise with stirring in an ice bath for 1 h. After completion of the reaction (monitored by TLC, eluent CHCl_3_ : ethanol 10:1, Rf**_1_** = 0.37, and Rf**_3_**=0.41), the formed precipitate was collected by filtration, washed several times with water, dried and recrystallized from ethanol.

- - 1. **4,6-Diamino-2-[(cyanomethyl)selenopyridine-3-carbonitrile (1):**

White crystals, yield 1.54 g (81%); mp.160-162 ºC, Lit. mp.158-160 ℃ [38]; FT-IR (ATR) *ν_max_*: 3357, 3316, 3240 (2NH_2_), 3032 (CH_arom._), 2974 (CH_aliph._), 2234, 2190 (2C≡N), 1637 (C=N) cm^-1^; ^1^H NMR (400 MHz, DMSO-*d_6_*): *δ* 6.48 (s, 2H, NH_2_ exchanged by D_2_O), 6.39 (s, 2H, NH_2_ exchanged by D_2_O), 5.53 (s, 1H, CH_pyridyl_), 4.06 (s, 2H, CH_2_) ppm; ^13^C NMR (100 MHz, DMSO-*d_6_*): *δ* 161.2, 157.3, 155.9, 119.8 (CN), 117.2 (CN), 85.9 (CH_pyridyl_), 82.7, 6.1 (CH_2_) ppm. Dept-135 NMR (100 MHz, DMSO-*d_6_*): *δ* 86.1 (CH_pyridyl_), 6.1 (CH_2_, exchangeable) ppm*. Anal*. Calcd. For C_8_H_7_N_5_Se (252.13): C, 38.11; H, 2.80; N, 27.78% Found: C, 38.28; H, 2.93; N, 27.63%.

- - 1. **4,6-Diamino-2-(methylselanyl)pyridine-3-carbonitrile (3)**:

White crystals, yield 1.43 g (83%); mp. 148-150 ºC, Lit. mp. 150-152 ℃ [38]; FT-IR (ATR) *ν_max_*: 3424, 3352, 3329, 3246 (2NH_2_), 2933 (CH_aliph._), 2203 (C≡N), 1623 (C=N) cm^-1^; ^1^H NMR (400 MHz, DMSO-*d_6_*): *δ* 6.30 (s, 2H, NH_2_), 6.19 (s, 2H, NH_2_), 5.47 (s, 1H, CH_pyridyl_), 2.35 (SeCH_3_) ppm; ^13^C NMR (100 MHz, DMSO-*d_6_*): *δ* 161.0, 158.9, 157.1, 117.9 (CN), 85.5 (CH_pyridyl_), 83.3, 5.7 (SeCH_3_) ppm. *Anal*. Calcd. For C_7_H_8_N_4_Se (227.12): C, 37.02; H, 3.55; N, 24.67%. Found: C, 37.16; H, 3.38; N, 24.81%.

- 1. **Synthesis of 3,4,6-triamino-2-cyanoselenopheno[2,3-*b*]pyridine (2):**

A mixture of selenopyridine **1** (1 g, 4 mmol) and TEA (0.4 g, 4 mmol) in 20 mL of ethanol was refluxed for 3 hrs. After completion of the reaction (monitored using TLC, eluent CHCl_3_ : ethanol 10:1, Rf = 0.18), the excess solvent was evaporated under vacuum. The resulting product was collected and recrystallized from ethanol.

Brown crystals yield 0.48 g (95%); mp. 258-260 ºC, Lit mp. 256-258 ℃ [38]; FT-IR (ATR) *ν_max_*: 3416, 3315, 3269, 3201 (3NH_2_), 2164 (C≡N), 1661 (C=N) cm^-1^; ^1^H NMR (400 MHz, DMSO-*d_6_*): *δ* 6.16 (s, 4H, 2NH_2_), 6.11 (s, 2H, NH_2_), 5.64 (s, 1H, CH) ppm; ^13^C NMR (100 MHz, DMSO-*d_6_*): *δ* 166.0, 161.0, 155.3, 153.8, 118.9 (CN), 107.5, 88.9 (CH), 62.8 ppm. *Anal*. Calcd. For C_8_H_7_N_5_Se (252.13): C, 38.11; H, 2.80; N, 27.78%. Found: C, 37.97; H, 2.92; N, 27.81%.

- 1. **Synthesis of 2,2'-diselenobis[4-amino-6-(dimethylamino)pyridine-3-carbonitrile (4):**

2-Aminoprop-1-ene-1,1,3-tricarbonitrile (1 g, 7.5 mmol ) in 50 mL of ethanol was added to a solution of sodium hydrogen selenide [freshly prepared from finely divided selenium powder (0.59 g, 7.5 mmol) and sodium borohydride (0.56 g, 15 mmol) in 20 mL of water] and the reaction mixture was refluxed under nitrogen conditions for 3 hrs., then cooled to 60℃, and methyl iodide (1 g, 7.5 mmol) was added dropwise with stirring for 1 h at 60 ^o^C. After completion of the reaction (monitored by TLC, eluent CHCl_3_ : ethanol 10:1, Rf =0.74), the formed precipitate was collected, washed with water, dried and recrystallized from ethanol.

Yellow powder, yield 1.1 g (31%); mp. 172-174 ºC. FT-IR (ATR) *ν_max_*: 3339, 3245 (NH_2_), 3043 (CH _arom._), 2918 (CH_aliph._), 2204 (C≡N), 1628 (C=N) cm^-1^; ^1^H NMR (400 MHz, DMSO-*d_6_*): *δ* 7.13 (s, 4H, 2NH_2_), 6.21 (s, 2H, 2CH_pyridyl_), 2.42 (s, 6H, N(CH_3_)_2_), 2.39 (s, 6H, N(CH_3_)_2_) ppm; ^13^C NMR (100 MHz, DMSO-*d_6_*): *δ* 160.3, 159.9, 149.1, 117.6 (CN), 101.9, 95.2 (CH_pyridyl_), 6.4 (2NCH_3_), 6.3 (2NCH_3_) ppm. *Anal*. Calcd. For C_16_H_18_N_8_Se_2_ (480.28): C, 40.01; H, 3.78; N, 23.33%. Found: C, 40.23; H, 3.62; N, 23.29%.

- 1. **Synthesis of 4,6-diamino-5-bromo-2-(cyanomethylselanyl)nicotinonitrile (5):**

To a solution of compound **1** (0.25 g, 2 mmol) in acetic acid (20 ml), bromine solution (0.31 g, 2 mmol) in acetic acid (5 ml) was added dropwislly with stirring for about 30 min at room temperature in the presence of sunlight. After completion of the reaction (monitored by TLC, eluent CHCl_3_ : ethanol 10:1, Rf =0.55), the formed precipitate was collected by filtration, washed by distilled water several times, dried and recrystallized from ethanol.

Brown crystal, yield 0.54 g (82%); mp. 176-178 ºC, FT-IR (ATR) *ν_max_*: 3470, 3412, 3344, 3232 (2NH_2_), 2982 (CH_aliph._), 2240, 2198 (2C≡N), 1644 (C=N) cm^-1^; ^1^H NMR (400 MHz, DMSO-*d_6_*): *δ* 6.92 (s, 2H, NH_2_), 6.64 (s, H, NH_2_), 4.08 (s, 2H, SeCH_2_) ppm; ^13^C NMR (100 MHz, DMSO-*d_6_*): *δ* 157.9, 154.8, 153.6, 119.6 (**C**N), 116.3 (**C**N), 83.3, 82.6, 6.8 (Se**C**H_2_) ppm. *Anal*. Calcd. For C_8_H_6_BrN_5_Se (331.03): C, 29.03; H, 1.83; N, 21.16%. Found: C, 29.21; H, 1.73; N, 21.32%.

- 1. **Synthesis of 3,4,6-triamino-5-bromoselenopheno[2,3-*b*]pyridine-2-carbonitrile (7):**

A mixture of selenopyridine **5** (1 g, 3 mmol) and TEA (0.3 g, 3 mmol) in 20 mL of ethanol was refluxed for 3 hrs. After completion of the reaction (monitored by TLC, silica gel, eluent CHCl_3_ : ethanol 10:1, Rf =0.29), the formed precipitate was filtrated and recrystallized from acetone.

Brown crystals, yield 0.86 g (86%); mp. 268-270 ºC; FT-IR (ATR) *ν_max_*: 3420, 3333, 3269 (3NH_2_), 3051 (CH_arom._) 2177 (C≡N) cm^-1^; ^1^H NMR (400 MHz, DMSO-*d_6_*): *δ* 6.51 (s, 2H, NH_2_), 6.36 (s, 2H, NH_2_), 6.15 (s, 2H, NH_2_) ppm; ^13^C NMR (100 MHz, DMSO-*d_6_*): *δ* 163.7, 156.9, 154.5, 149.9, 118.2 (CN), 108.0, 86.3, 67.2 ppm. *Anal*. Calcd. For C_8_H_6_BrN_5_Se (331.03): C, 29.03; H, 1.83; N, 21.16%. Found: C, 29.12; H, 1.72; N, 21.21%.

- 1. **General procedure for synthesis of compounds 8a,b:**

A mixture of compound **5** (0.5 g, 1.5 mmol), and (1.5 mmol) an appropriate thiol namely: thiophenol (0.17 g, 1.5 mmol (**Method A**) and/or 0.34 g, 3 mmol (**Method B**) and/or *p*-chlorothiophenol (0.22 g, 1.5 mmol (**Method A**) and/or 0.44 g, 3 mmol (**Method B**) with a catalytic amount of TEA dissolved in ethanol (30 mL) and refluxed for 8 hrs. After completion of the reaction (monitored by TLC, eluent CHCl_3_ : ethanol 10:1, Rf**_8a_** = 0.70, Rf**_8b_** = 0.74), the excess solvent was evaporated under vacuum. The resulting product was collected and recrystallized from the appropriate solvent.

- - 1. **2,2'-Diselanediylbis(4,6-diamino-5-(phenylthio)pyridine-3-carbonitrile) (8a):**

Brown crystal (ethanol); yield (30% Method **A**, 62% Method **B**); mp. 198-200ºC ºC; FT-IR (ATR) *ν_max_*: 3450, 3335, 3304 (2NH_2_), 3079 (CH_arom._), 2236 (C≡N), 1617 (C=N) cm^-1^; ^1^H NMR (400 MHz, DMSO-*d_6_*): *δ* 7.50-7.47 (m, 4H, CH_arom._), 7.43-7.39 (m, 6H, CH_arom._), 6.62 (s, 4H, 2NH_2_), 6.51 (s, 4H, 2NH_2_) ppm; ^13^C NMR (100 MHz, DMSO-*d_6_*): *δ* 159.5, 157.8, 153.7, 133.8, 130.6, 129.7, 128.9, 116.1 (CN), 83.3, 82.9 ppm. *Anal*. Calcd. For C_24_H_18_N_8_S_2_Se_2_ (640.50): C, 45.00; H, 2.83; N, 17.49%. Found: C, 45.11; H, 2.91; N, 17.40%.

- - 1. **2,2'-Diselanediylbis(4,6-diamino-5-(4-chlorophenylthio)pyridine-3-carbonitrile) (8b):**

Yellow crystal (acetone); yield (33% Method **A**, 70% Method **B**); mp. 236-238 ºC; FT-IR (ATR) *ν_max_*: 3453, 3337, 3302 (2NH_2_), 3085 (CH_arom._), 2236 (C≡N), 1617 (C=N) cm^-1^; ^1^H NMR (400 MHz, DMSO-*d_6_*): *δ* 7.52-7.45 (dd, 8H, *J* = 19, 8 Hz, CH_arom._), 6.59 (s, 4H, 2NH_2_ exchanged by D_2_O), 6.46 (s, 4H, 2NH_2_ exchanged by D_2_O) ppm; ^13^C NMR (100 MHz, DMSO-*d_6_*): *δ* 159.1, 157.8, 153.7, 135.7, 133.9, 129.6, 129.4, 115.9 (CN), 82.9, 82.8 ppm; Dept-135 NMR (100 MHz, DMSO-*d_6_*): *δ* 135.7 (CH_arom_), 129.6 (CH_arom_) ppm. *Anal*. Calcd. For C_24_H_16_C_l2_N_8_S_2_Se_2_: (711.39): C, 40.63; H, 2.27; N, 15.80%. Found: C, 40.70; H, 2.19; N, 15.68%.

- 1. **Synthesis of 2-(benzo[*d*]thiazol-2-yl)-5-bromo-selenopheno[2,3-*b*]-pyridine-3,4,6-triamine (9):**

A mixture of compound **5** (1 g, 3 mmol) and *o-*aminothiophenol (0.377 g, 3 mmol was refluxed in ethanol (30 mL) for 6 hrs) in the presence of catalytic amount of TEA. After completion of the reaction (monitored by TLC, eluent CHCl_3_ : ethanol 10:1, Rf = 0.59), the formed precipitate was filtrated and recrystallized from acetone.

Yellow crystal, yield 0.45 g (68%); mp. dec. 270-272 ºC; FT-IR (ATR) *ν_max_*: 3463, 3426, 3348 (3NH_2_), 3071 (CH_arom._), 1623 (C=N) cm^-1^; ^1^H NMR (400 MHz, DMSO-*d_6_*): *δ* 7.26 (d, 1H, *J* = 8 Hz, CH_arom._), 7.15 (t, 1H, *J*= 7 Hz, CH_arom._), 6.77 (d, 1H, *J* = 8 Hz, CH_arom._), 6.57 (t, 1H, *J* = 7 Hz, CH_arom._), 6.50 (s, 2H, NH_2_), 6.45 (s, 2H, NH_2_), 5.28 (s, 2H, NH_2_) ppm; ^13^C NMR (100 MHz, DMSO-*d_6_*): *δ* 160.3, 157.6, 153.6, 151.2, 137.6, 131.5, 116.9, 116.3, 115.6, 110.7, 82.4, 82.3 ppm. *Anal*. Calcd. For C_14_H_10_BrN_5_SSe (439.19): C, 38.29; H, 2.29; N, 15.95% Found: C, 38.40; H, 2.19; N, 15.89%.

- 1. **Synthesis of *N*-(2-cyano-4-methyl-5*H*-1-seleno-3,5,8-triazaacenaphthylen-7-yl)acetamide (11):**

Selenopheno[2,3-*b*]pyridine **1** (0.5 g, 2 mmol) was refluxed in 15 mL of acetic anhydride for 3 hours and allowed to cool at room temperature, then poured into 50 mL of cold water and left to stand for 1 h. After completion of the reaction (monitored by TLC, eluent CHCl_3_ : ethanol 10:1, Rf = 0.25), the formed precipitate was filtered off, washed with distilled water several times, dried and recrystallized from ethanol.

White powder, yield 0.49 g (78%); mp. ˃300 ºC; FT-IR (ATR) *ν_max_*: 3249, 3162, (2NH), 3013 (CH_arom._), 2971 (CH_aliph._), 2180 (C≡N), 1673 (C=O) cm^-1^; ^1^H NMR (400 MHz, DMSO-*d_6_*): *δ* 11.87 (s, 1H, NH exchanged by D_2_O), 10.79 (s, 1H, NH exchanged by D_2_O), 7.62 (s, 1H, CH-6_._), 2.23 (s, 3H, CO**CH_3_**), 2.11 (s, 3H, CH_3_) ppm; ^13^C NMR (100 MHz, DMSO-*d_6_*): *δ* 170.2 (C=O), 160.6, 158.9, 156.1, 155.5, 151.8, 145.6, 116.7 (CN), 116.2, 92.0, 24.6 (CO**CH_3_**), 22.4 (CH_3_) ppm. *Anal*. Calcd. For C_12_H_9_N_5_OSe (318.19): C, 45.30; H, 2.85; N, 22.01% Found: C, 45.39; H, 2.74; N, 22.09%.

- 1. **General procedure for synthesis of 3,4,6-Triamino-5-[aryldiazenyl]selenopheno[2,3-*b*]pyridine-2-carbonitrile 12a-d:**

Sodium nitrite (0.14 g, 2 mmol) in 2 mL of cold water was added slowly at 0-5 ºC to a stirred solution of appropriate aromatic amines (2 mmol) namely aniline (0.18 g), *p*-toluidine (0.21 g), *p*-methoxyaniline (0.24 g) and *p*-chloroaniline (0.25 g) in 5 mL of conc. HCl. The formed diazonium salt solution was added with continuous stirring to an ice cooled solution of selenopheno[2,3-*b*]pyridine **2** (0.5 g, 2 mmol) in 20 mL of pyridine at 0-5 ºC. The reaction mixture was allowed to stand for 30 minutes. (in an ice bath) and then poured into 50 mL cold water. After completion of the reaction (monitored by TLC, eluent CHCl_3_ : ethanol 10:1, **12a-d**, Rf = 0.40, 0.48, 0.37, 0.40, respectively), the formed precipitate filtered off, washed with distilled water several times, dried and recrystallized from dioxane.

- - 1. **3,4,6-Triamino-5-[phenyldiazenyl]selenopheno[2,3-*b*]pyridine-2-carbonitrile (12a):**

Beige powder, yield 0.6 g (85%); mp. dec 240-242 ºC; FT-IR (ATR) *ν_max_*: 3399, 3369, 3309, 3212 (3NH_2_), 3022 (CH_arom._), 2211 (C≡N), 1628 (C=N) cm^-1^ ; ^1^H NMR (400 MHz, DMSO-*d_6_*): *δ* 11.38 (s, 1H, NH), 7.43-7.35 (m, 4H, CH_arom._), 7.07 (t, 1H,  *J* = 7 Hz, CH_arom._) 6.97 (s, 2H, NH_2_), 6.38 (s, 2H, NH_2_), 5.71 (s, 1H, NH) ppm.^13^C NMR (100 MHz, DMSO-*d_6_*): *δ* 180.1, 160.5, 160.1, 153.6, 143.2, 129.9, 123.6, 115.6 (CN), 115.0, 112.5, 111.3, 87.4 ppm. UV-Vis (λ_max_, nm, DMSO): 378 nm. *Anal*. Calcd. For C_14_H_11_N_7_Se (356.24): C, 47.20; H, 3.11; N, 27.52%. Found: C, 47.36; H, 3.32; N, 27.74%.

- - 1. **3,4,6-Triamino-5-(4-methylphenyl)diazenylselenopheno[2,3-*b*]pyridine-2-carbonitrile (12b):**

Yellow powder, yield 0.65 g (89%); mp. dec. 220-222 ºC; FT-IR (ATR) *ν_max_*: 3383, 3300, 3173 (3NH_2_), 3017 (CH_arom._), 2962 (CH_aliph._), 2213 (C≡N), 1647 (C=N) cm^-1^; ^1^H NMR (400 MHz, DMSO-*d_6_*): *δ* 11.32 (s, 1H, NH), 7.30 (d, 2H, *J* = 8 Hz, CH_arom._), 7.17 (d, 2H, *J* = 8 Hz, CH_arom._), 6.98 (s, 2H, NH_2_), 6.35 (s, 2H, NH_2_), 5.69 (s, 1H, NH), 2.27 (s, 3H, CH_3_) ppm; ^13^C NMR (100 MHz, DMSO-*d_6_*): *δ* 180.1, 160.5, 160.2, 153.6, 140.9, 132.8, 130.3, 115.7 (CN), 114.4, 112.6, 111.4, 87.4, 20.8 (CH_3_) ppm; UV-Vis (λ_max_, nm, DMSO): 380 nm. *Anal*. Calcd. For C_15_H_13_N_7_Se (370.27): C, 48.66; H, 3.54; N, 26.48%. Found: C, 48.51; H, 3.73; N, 26.65%.

- - 1. **3,4,6-Triamino-5-[(4-methoxyphenyl)diazenyl]selenopheno[2,3-*b*]pyridine-2-carbonitrile (12c):**

Reddish brown powder, yield 0.66 g (87%); mp. dec. 278-280 ºC; FT-IR (ATR) *ν_max_*: 3399, 3302, 3173 (3NH_2_), 3042 (CH_arom._), 2836 (CH_aliph._), 2210 (C≡N), 1658 (C=N) cm^-1^; ^1^H NMR (400 MHz, DMSO-*d_6_*): *δ* 11.37 (s, 1H, NH ), 7.53 (s, 2H, NH_2_), 7.34 (d, 2H, *J* = 8 Hz, CH_arom._), 7.02 (s, 2H, NH_2_), 6.96 (d, 2H, *J* = 8 Hz, CH_arom._), 5.74 (s, 1H, NH), 3.75 (s, 3H, OCH_3_) ppm; ^13^C NMR (100 MHz, DMSO-*d_6_*): *δ* 175.8, 159.7, 157.9, 156.3, 154.3, 136.6, 117.3 (CN), 115.2, 113.4, 112.6, 112.1, 87.1, 55.8 (OCH_3_) ppm; UV-Vis (λ_max_, nm, DMSO): 382 nm. *Anal*. Calcd. For C_15_H_13_N_7_OSe (386.27): C, 46.64; H, 3.39; N, 25.38%. Found: C, 46.72; H, 3.28; N, 25.47%.

- - 1. **3,4,6-Triamino-5-[(4-chlorophenyl)diazenyl]selenopheno[2,3-*b*]pyridine-2-carbonitrile (12d):**

Dark yellow powder, yield 0.7 g (92%); mp. dec. 246-248 ºC; FT-IR (ATR) *ν_max_*: 3323, 3242, 3202 (3NH_2_), 3060 (CH_arom._), 2215 (C≡N), 1616 (C=N) cm^-1^; ^1^H NMR (400 MHz, DMSO-*d_6_*): *δ* 11.46 (br, 1H, NH), 7.41 (s, 4H, CH_arom._), 6.81 (s, 2H, NH_2_), 6.36 (s, 2H, NH_2_), 5.68 (s, 1H, NH) ppm; ^13^C NMR (100 MHz, DMSO-*d_6_*): *δ* 179.8, 160.4, 159.9, 153.5, 142.2, 129.7, 127.2, 117.2 (CN), 115.3, 112.4, 111.3, 87.5 ppm; UV-Vis (λ_max_, nm, DMSO): 378 nm. *Anal*. Calcd. For C_14_H_10_ClN_7_Se (390.68): C, 43.04; H, 2.58; N, 25.10%. Found: C, 43.19; H, 2.42; N, 25.27%.

1. **Biology**
   1. **Anticancer Activity**
      1. **Cell Lines and Culture Conditions**

The human prostate cancer cells (PC3) and human osteosarcoma cells (MG-63) were obtained from Nawah Scientific Inc. (Mokatam, Cairo, Egypt) and cultured in DMEM (Dulbecco’s modified eagle’s medium), Gibco, USA, supplemented with fetal bovine serum (FBS) at a concentration of 10% and 100 U/mL of penicillin and streptomycin (PS). The Cells were incubated at 37 °C in a humidified environment that contained 5% CO_2_.

- - 1. **Assessment of Cytotoxicity by SRB assay**

Cells were seeded in 96-well plates as aliquots of 100 μL cell suspension and incubated in a complete media for 24 h. Afterward, cells were treated with synthesized compounds at diverse concentrations ranging from 0.01 μM to 100 μM for 72 h. Then, cells were fixed at 4 °C for one hour with 150 μL of 10% trichloroacetic acid (TCA). After washing the cells five times with distilled water, 70 μL of sulforhodamine (SRB) solution (0.4% w/v) was added and incubated for 10 min at room temperature in a dark place. Cells were washed with 1% acetic acid three times and allowed to air-dry. Then, 150 μL of Tris pH 10.5 (10 mM) was added, and the absorbance was measured at 540 nm using a BMG LABTECH®- FLUOstar Omega microplate reader (Ortenberg, Germany) [52]. Half maximal inhibitory concentrations (IC_50_) values were calculated for each experiment using GraphPad Prism 6 software. IC_50_ values were reported as mean ± SD [53].

- - 1. **EGFR inhibitory assay**

A cell-free assay was used to investigate the mechanism of inhibition of EGFR kinase according to the reported method [54]. Kit used for immune-assay was cloud clone SEA757Hu 96 Tests. 200 lM (EGFR) was used. From the following equation: E (%) = E max/(1 + [I]/ID50), where E (%) is the fraction of the enzyme activity measured in the presence of the inhibitor, E max is the activity in the absence of the inhibitor, [I] is the inhibitor concentration and ID50 is the inhibitor concentration at which E (%) = 0.5 E max, a dose–response curve was generated. Mean values of two independent replicates for each experiment were used for the interpolation.

- - 1. **Statistical analysis**

Computerized Prism 5 program was used to statistically analyzed data using one-way ANOVA test followed by Tukey’s as post ANOVA for multiple comparison at P ≤.05. Data were presented as mean ± SEM.

- 1. **Docking study**

The 3.5 Å3D structure of EGFR (PDB ID: 1M17) [54] in complex with AQ4999 was downloaded from protein data bank. All molecular modeling calculations and docking studies were carried out using Discovery Studio software 2016 client v16.1.0.15350 (San Diego, CA) with CDOCKER program. Removing of chains A, B and E of the protein together with co-crystallized water molecules was performed. Automatic protein preparation module was used applying CHARMm force field. The binding site sphere has been defined automatically by the software. The docked compounds were built using Chem. 3D ultra 12.0 software [Chemical Structure Drawing Standard; Cambridge Soft corporation, USA (2010)], and copied to Discovery Studio 2016 client v16.1.0.15350. Ligands were prepared using “Prepare Ligands” protocol in Discovery Studio where hydrogen atoms were added at their standard geometry, optical isomers and 3D conformations were automatically generated. Docking was performed using CDOCKER protocol in Discovery Studio keeping the parameters at default. Each compound would retain 10 poses and the best scoring pose of the docked compounds was recognized. Receptor–ligand interactions of the complexes were examined in 2D and 3D styles.

# Copies of IR, ^1^H, D_2_O, ^13^CNMR and Dept-135 spectra:

- 1. **IR, ^1^H (D_2_O), ^13^C NMR and Dept-135 Spectra of 1:**


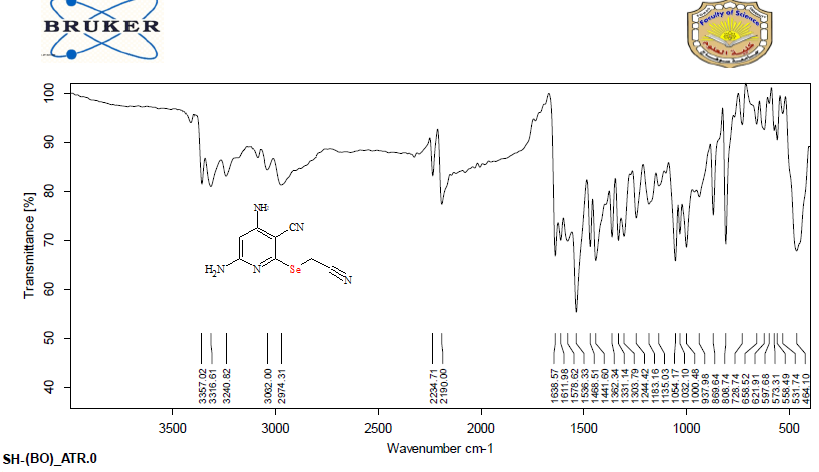


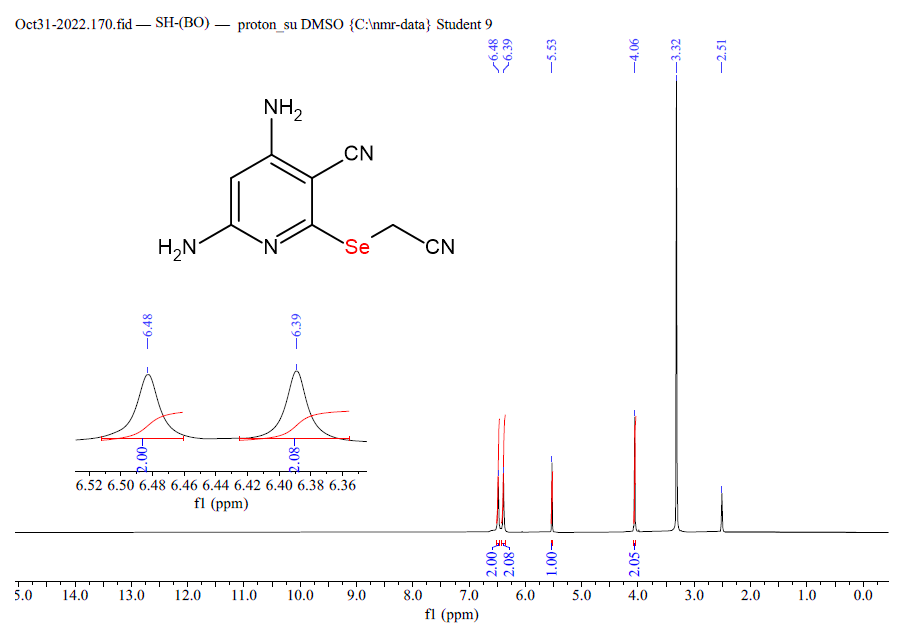


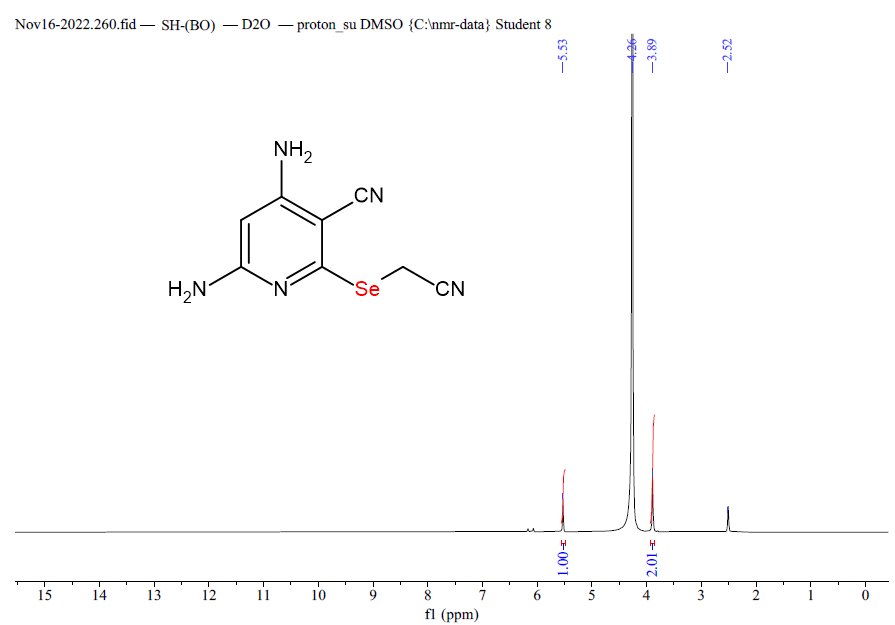


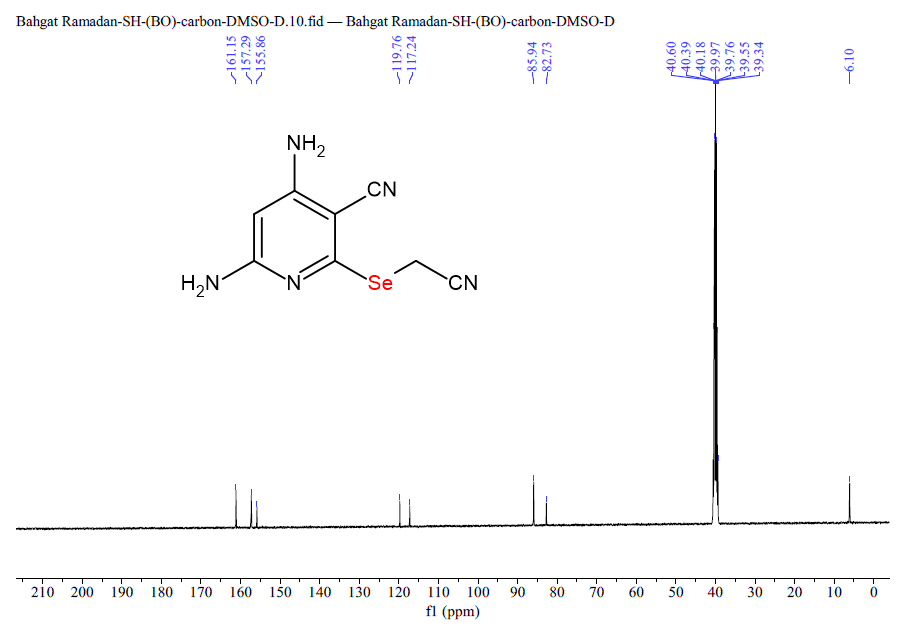


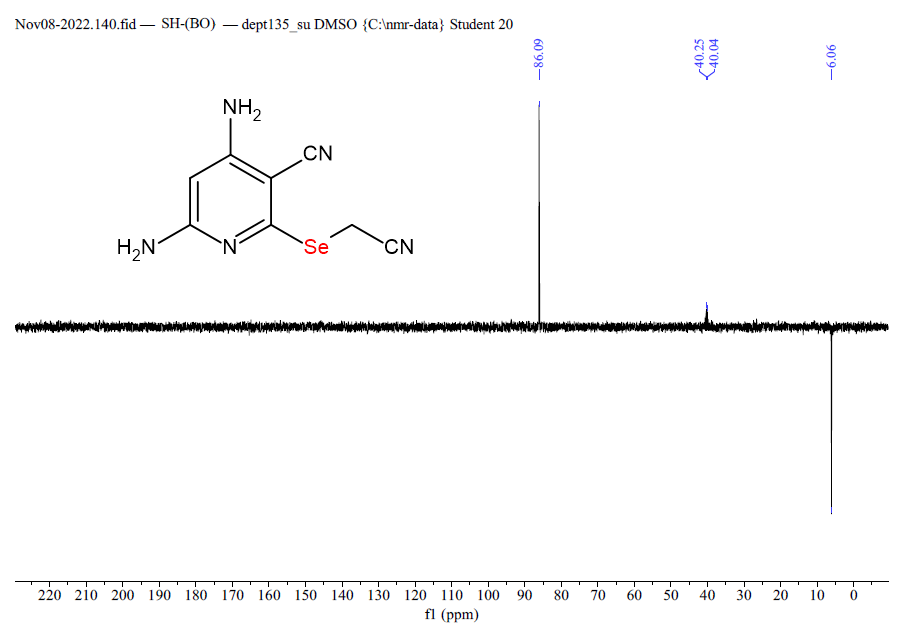


- 1. **IR, ^1^H and ^13^C NMR Spectra of 2:**

**
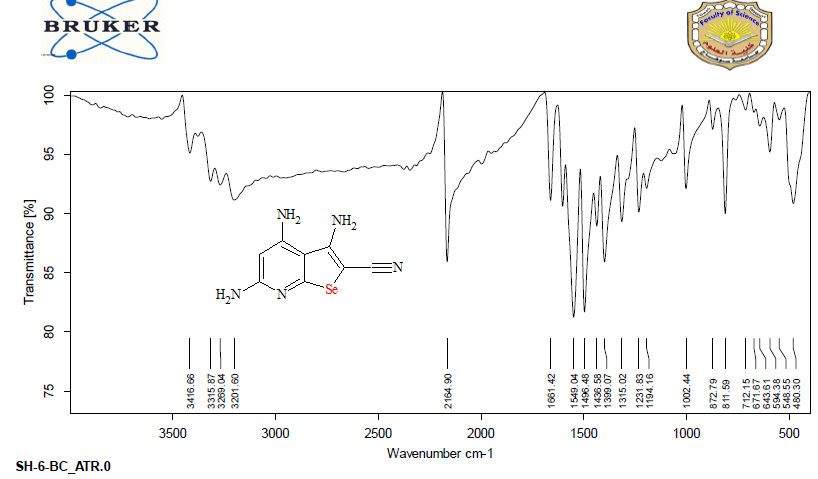
**


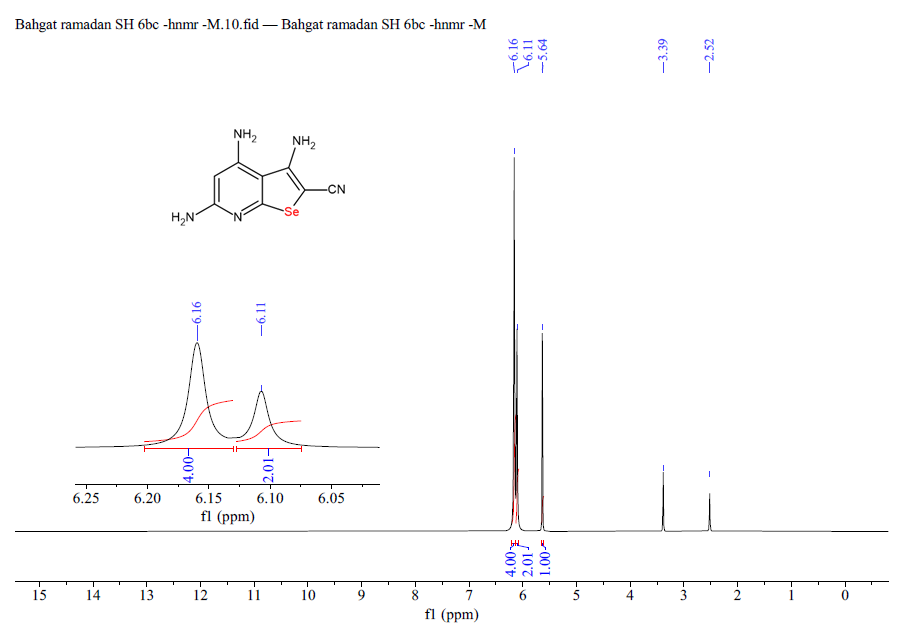


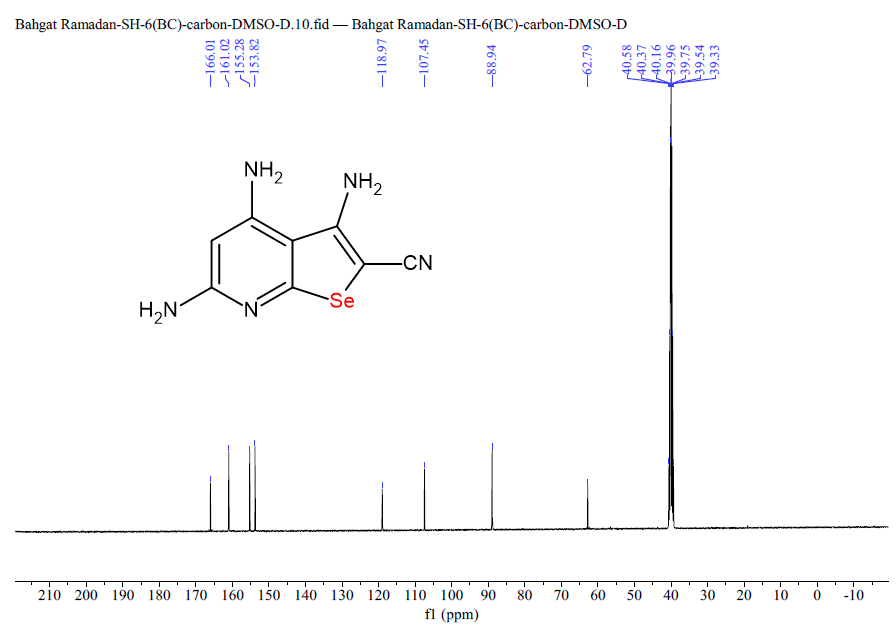


- 1. **IR, ^1^H and ^13^C NMR Spectra of 3:**

**
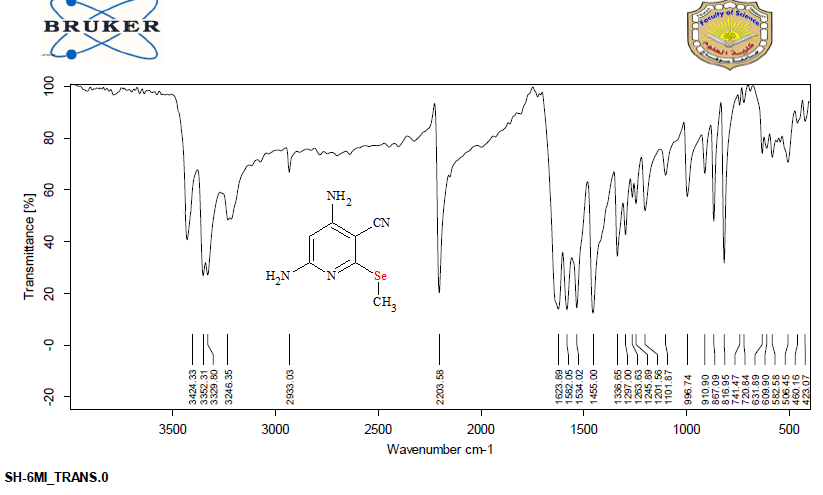
**


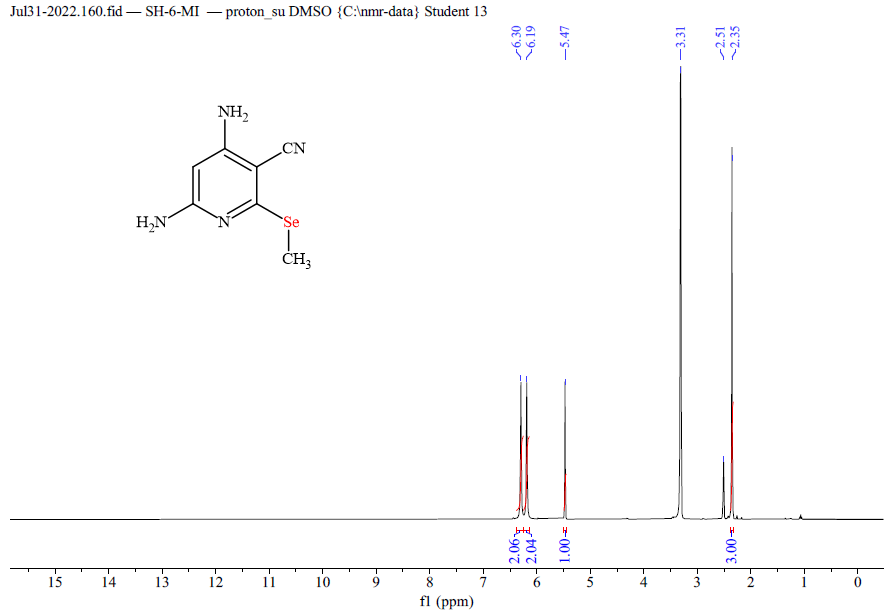


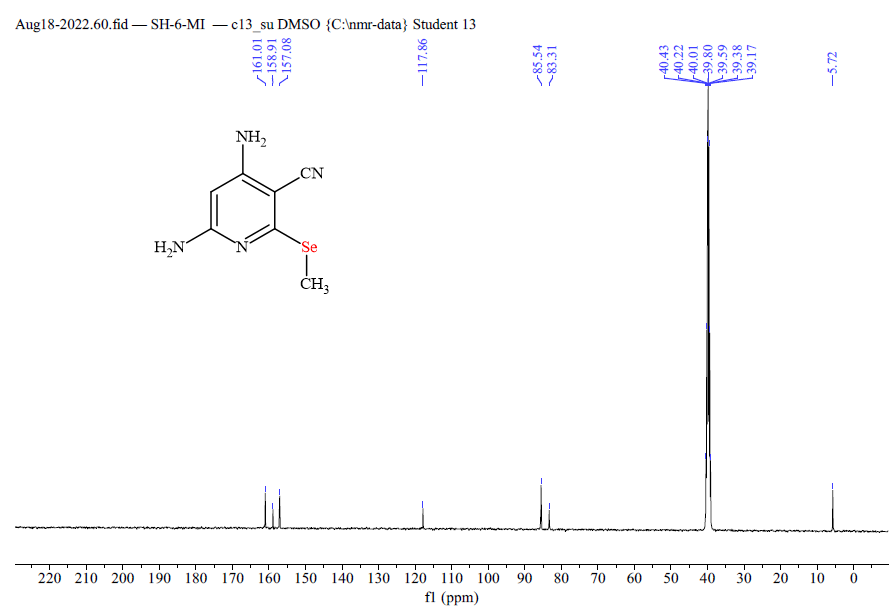


- 1. **IR, ^1^H, ^13^C NMR Spectra and X-RD analysis of 4:**

**
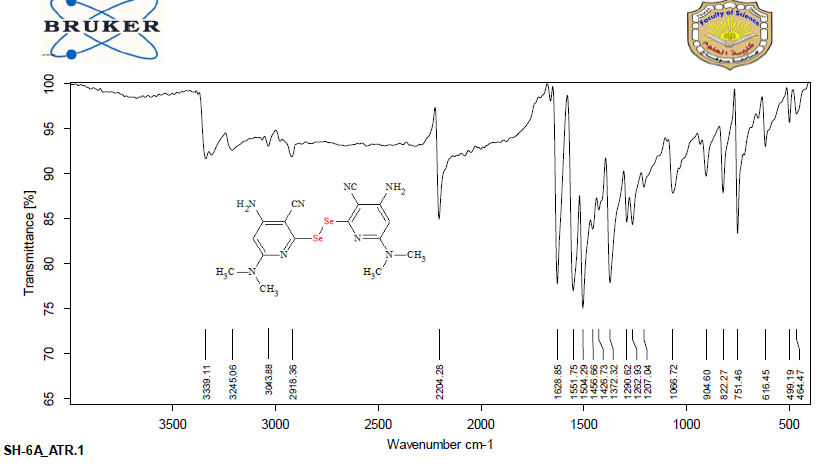
**

**
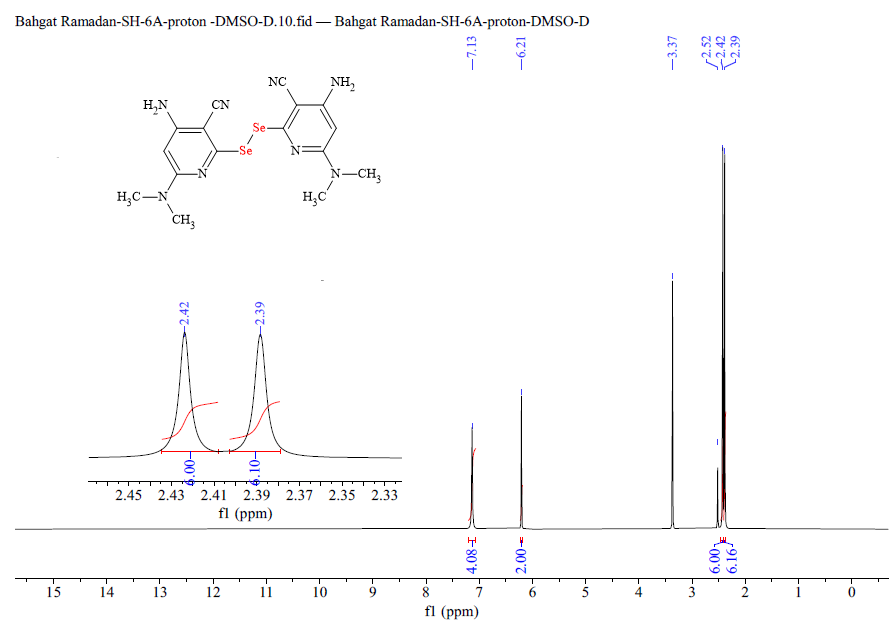
**

**
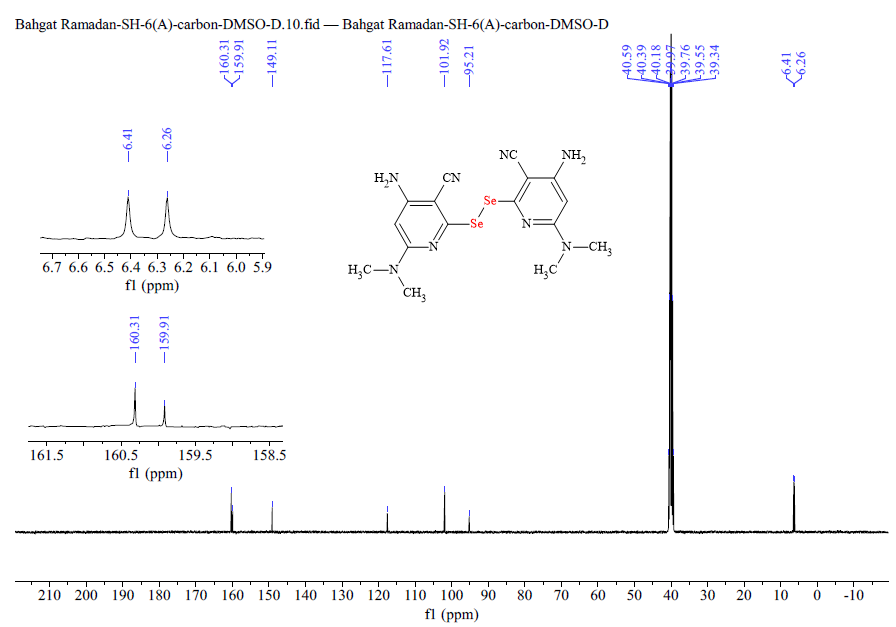
**

**
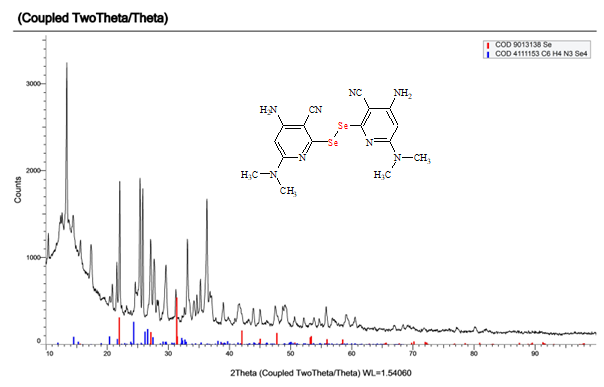
**

- 1. **IR, ^1^H and ^13^C NMR Spectra of 5:**

**
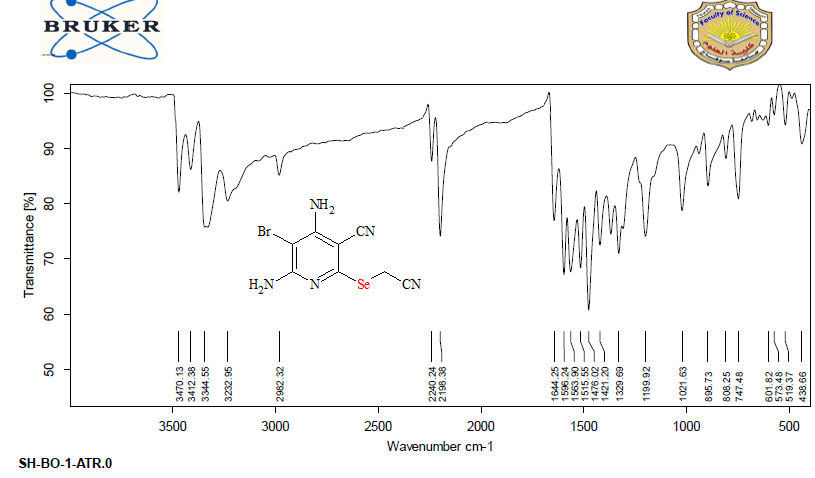
**


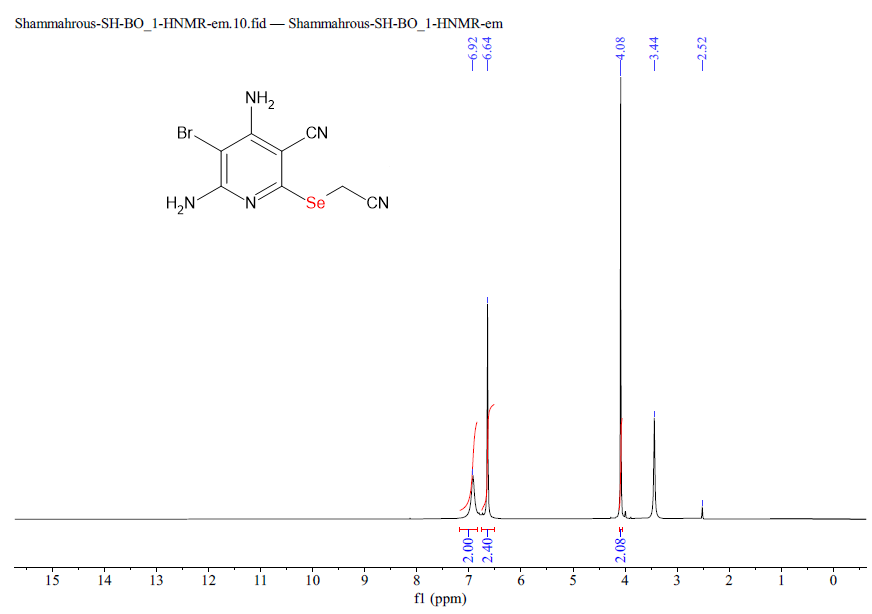


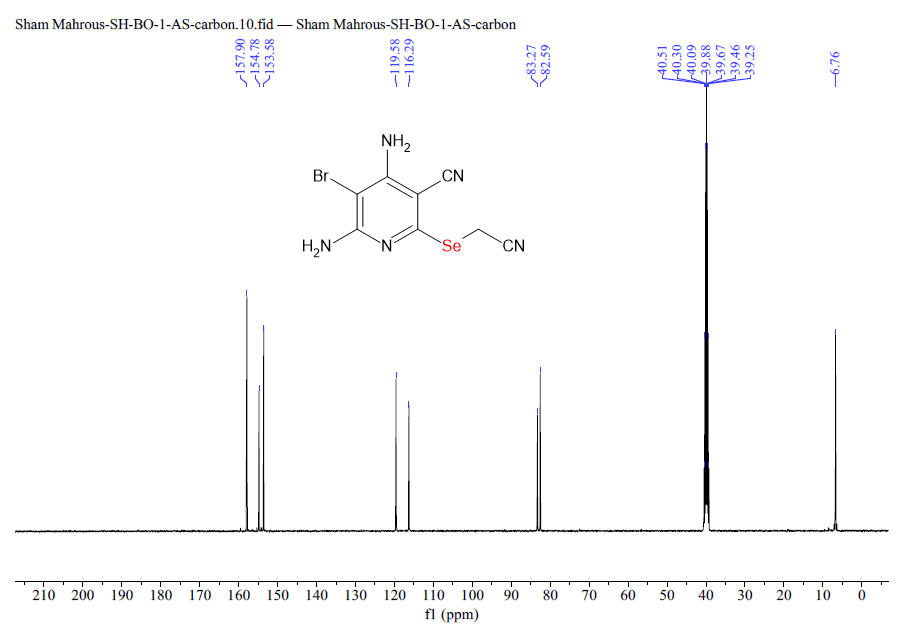


- 1. **IR, ^1^H and ^13^C NMR Spectra of 7**

:


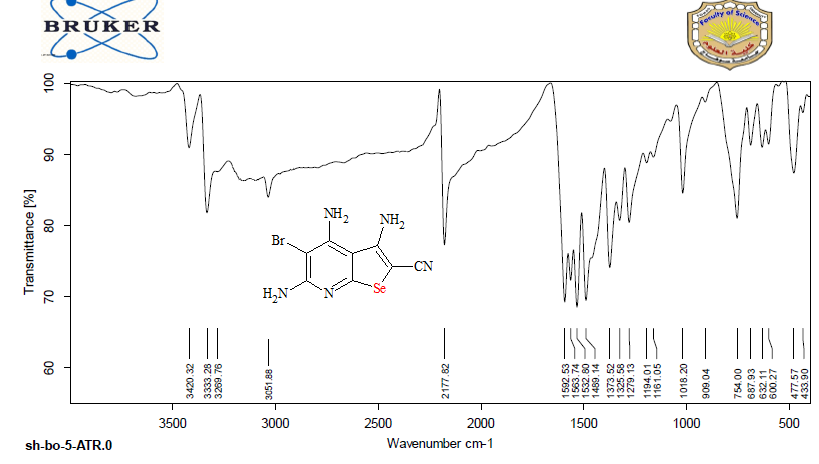


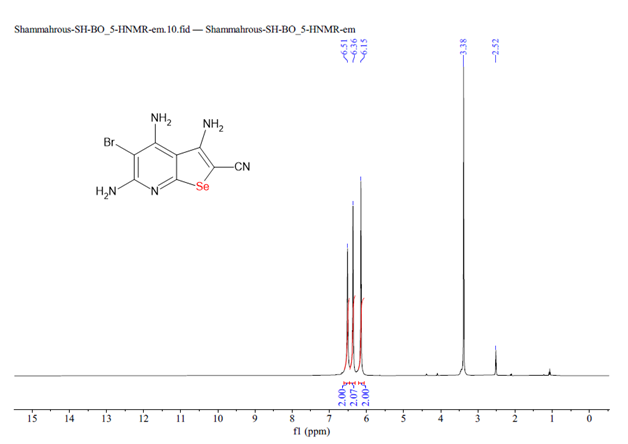


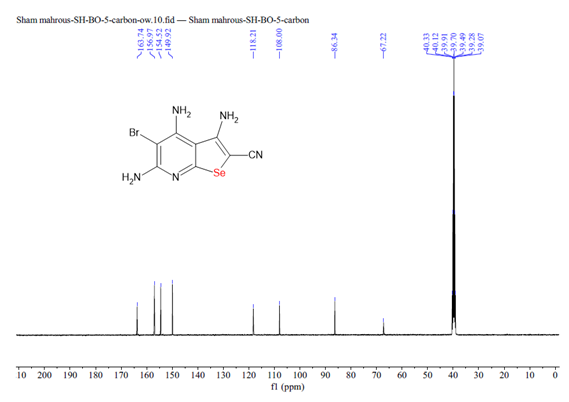


- 1. **IR, ^1^H and ^13^C NMR Spectra of 8a:**

**
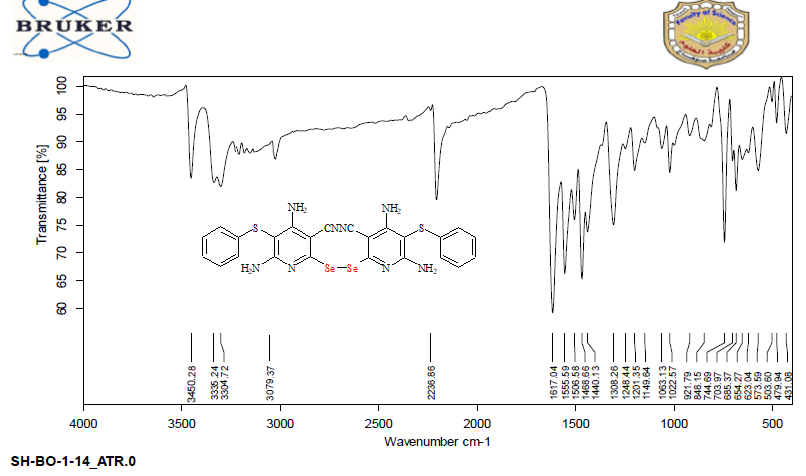
**


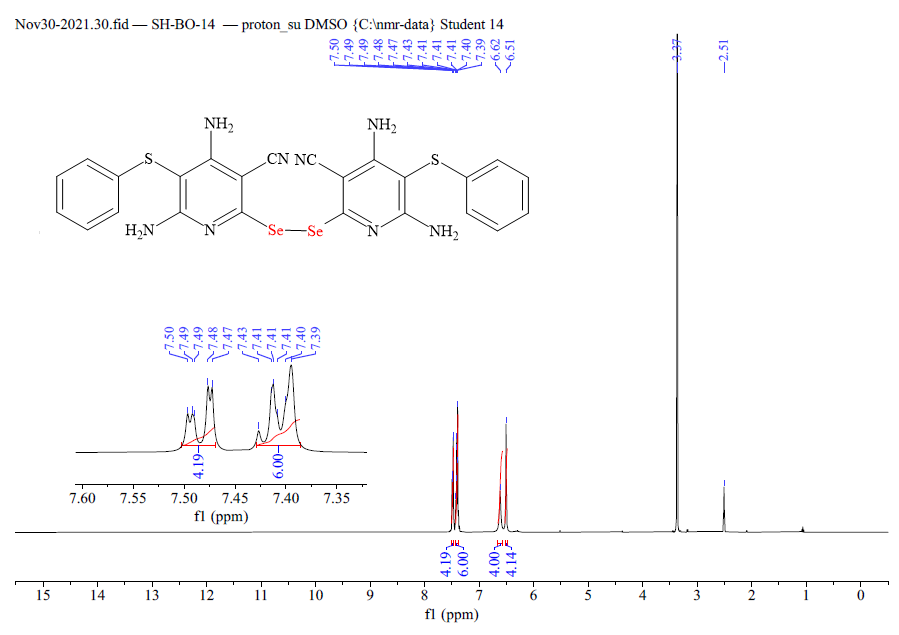


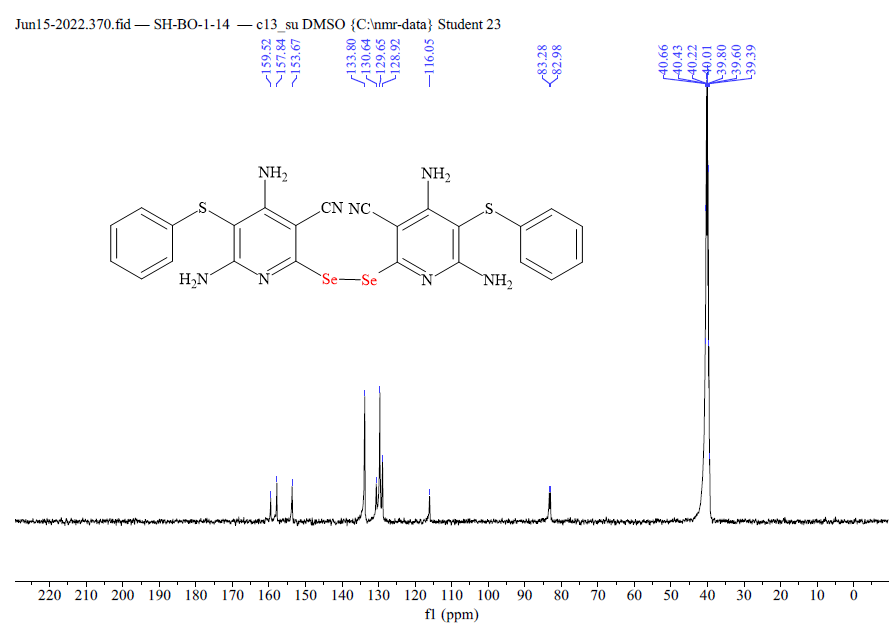


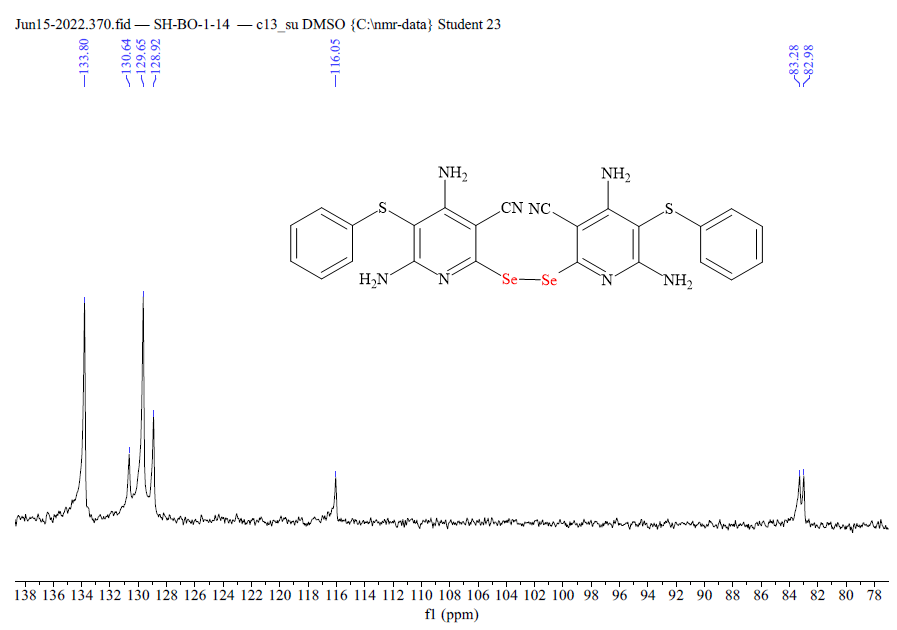


- 1. **IR, ^1^H (D_2_O), ^13^C NMR, Dept-135 Spectra and X-RD analysis of 8b:**

**
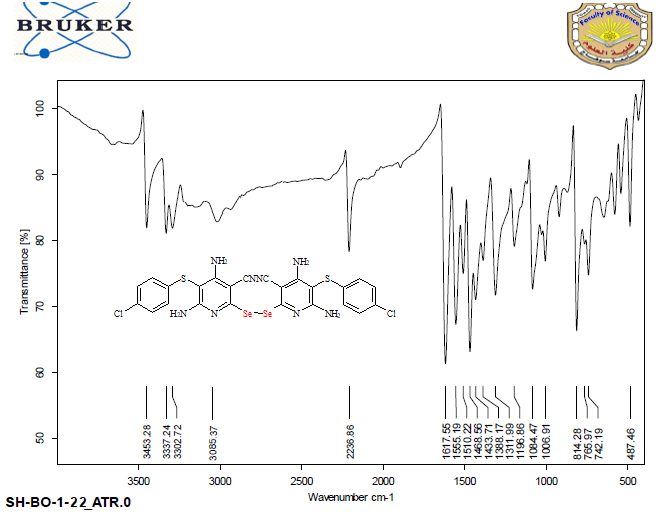
**


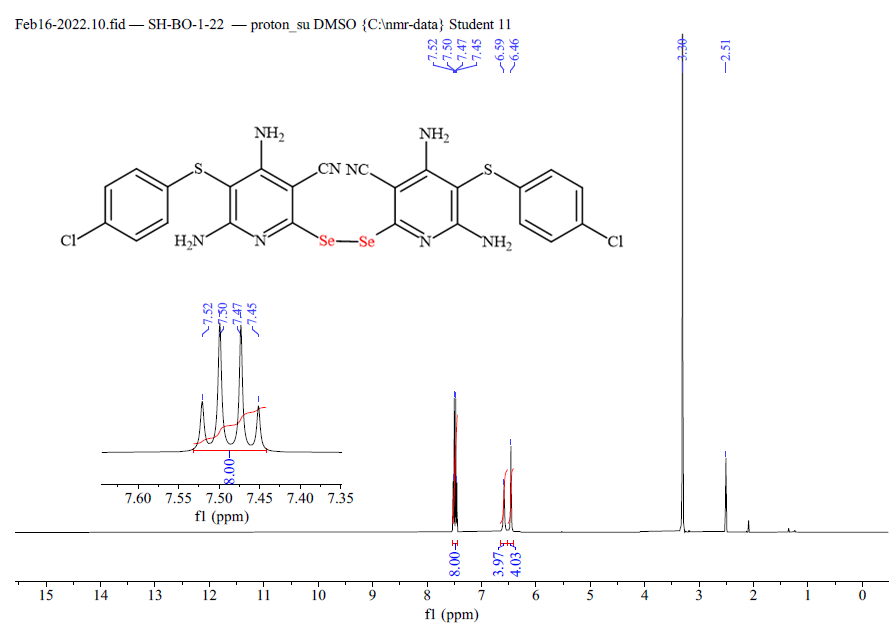


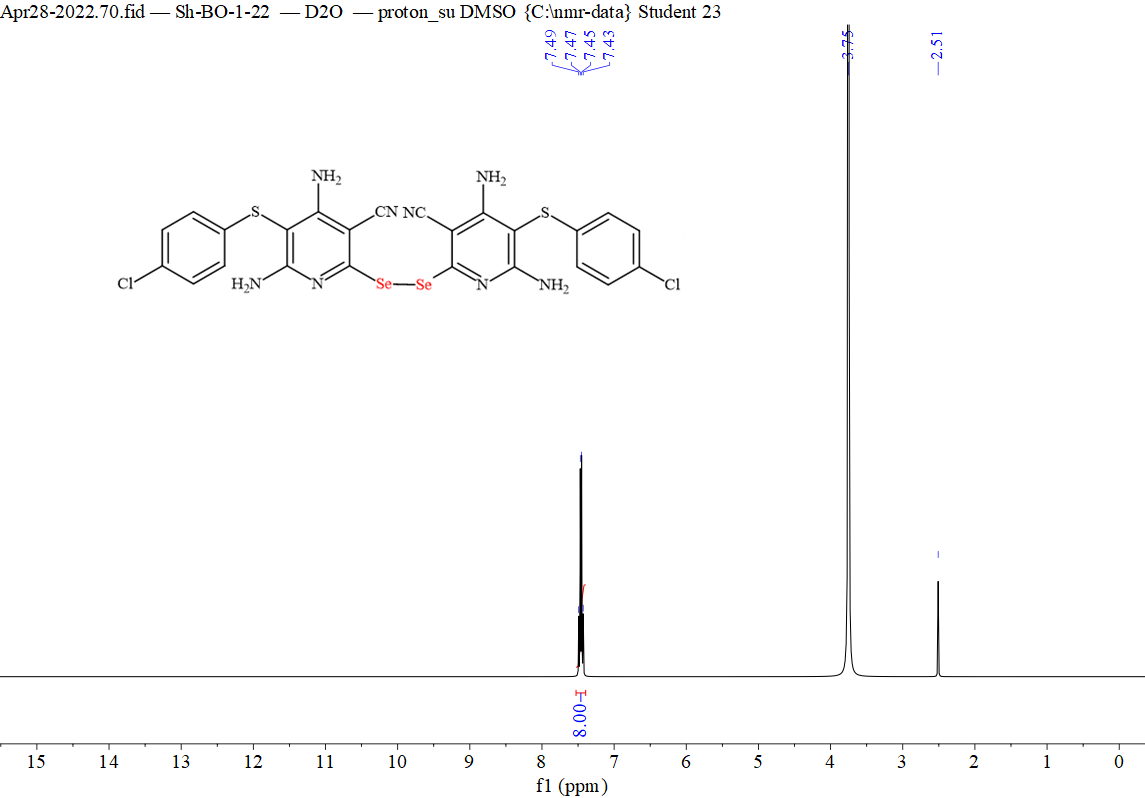


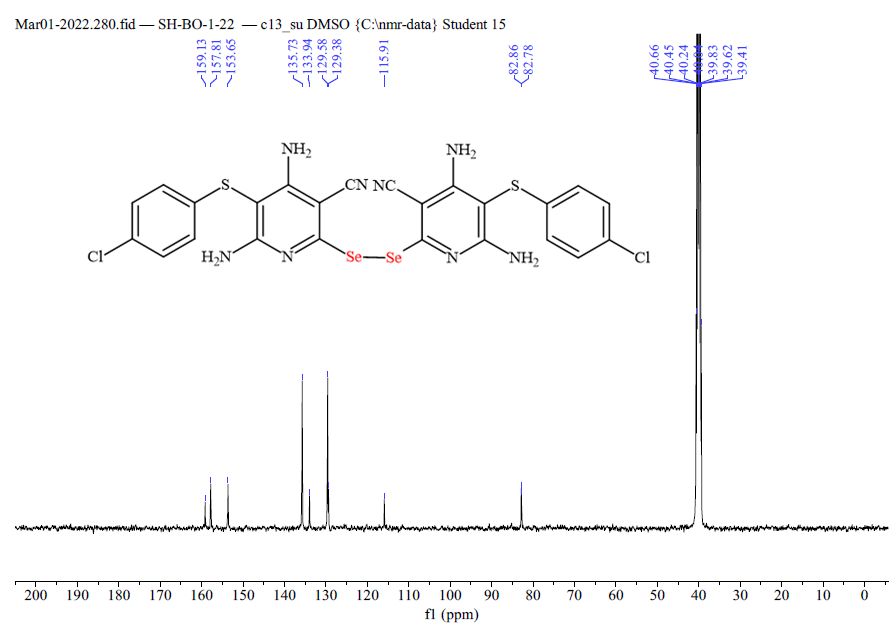


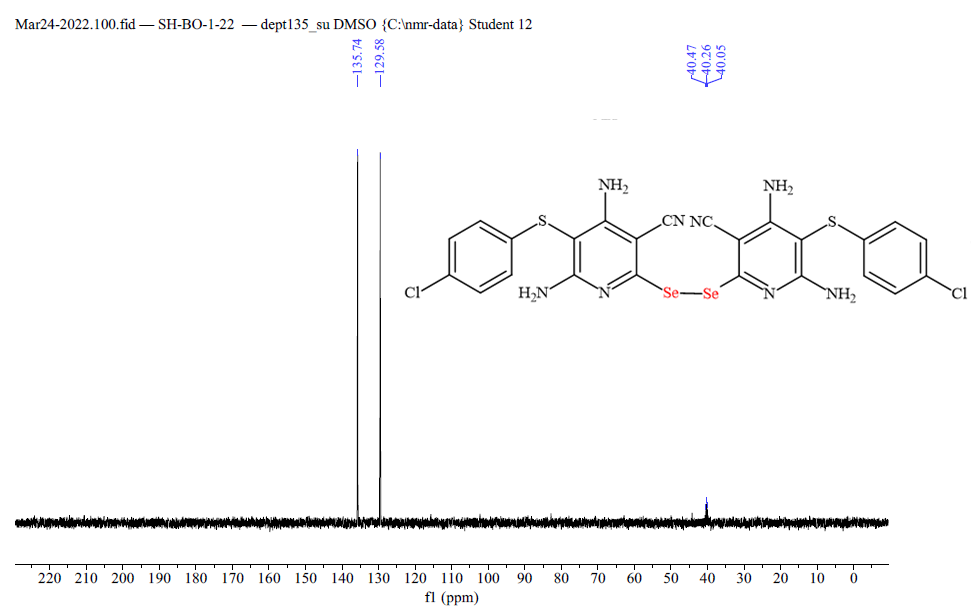


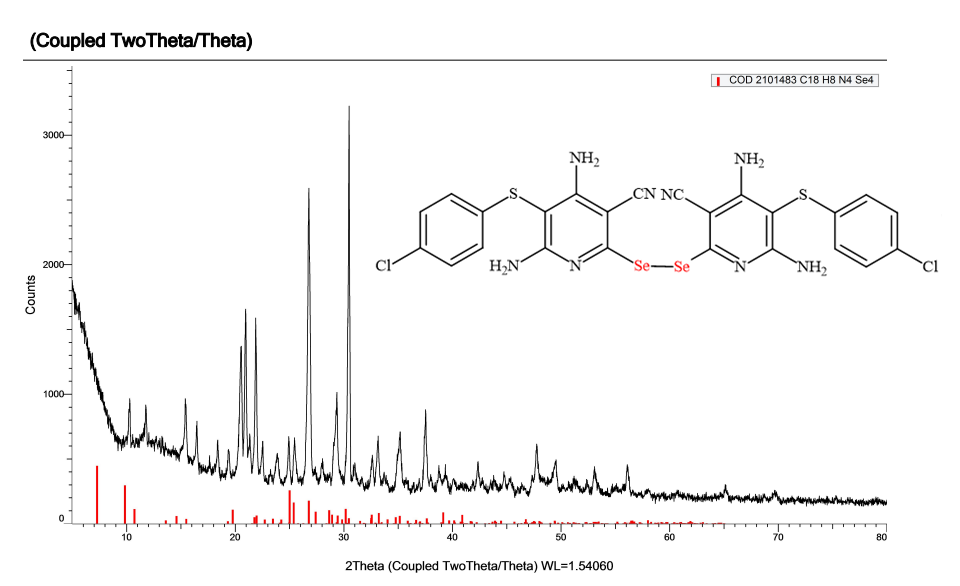


- 1. **IR, ^1^H and ^13^C NMR Spectra of 9:**


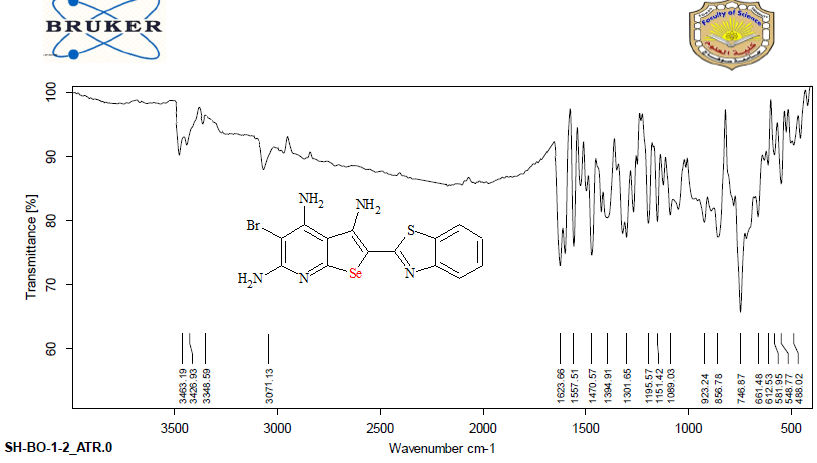


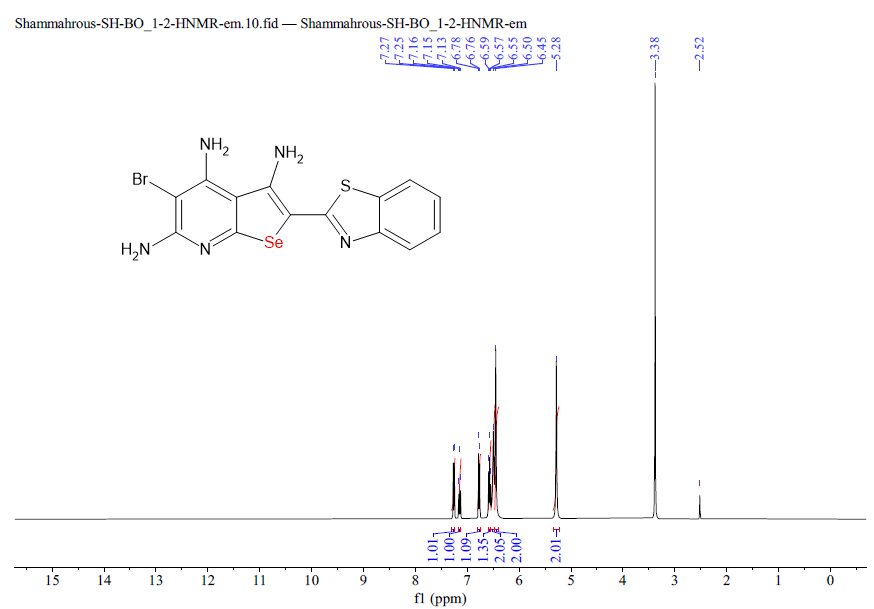


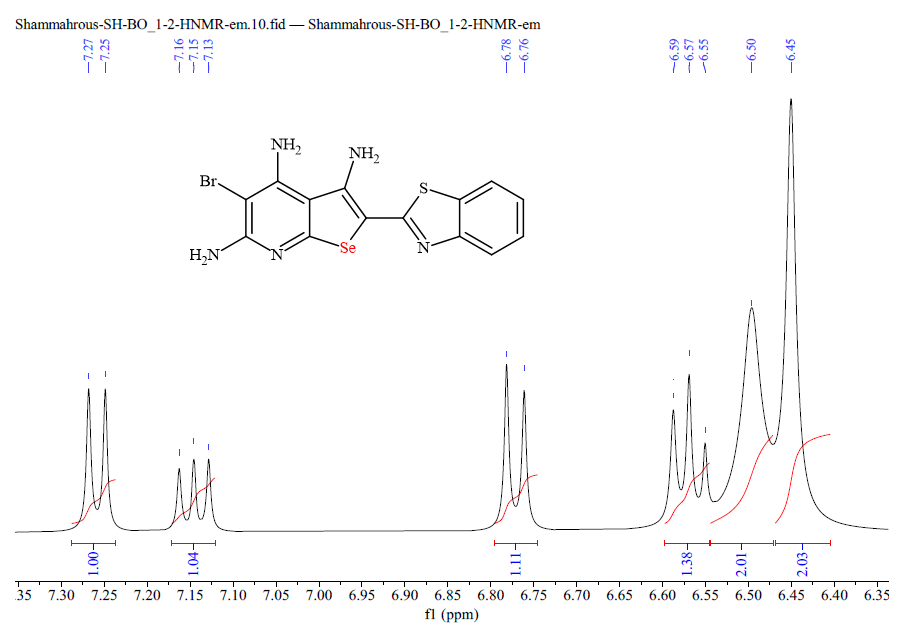


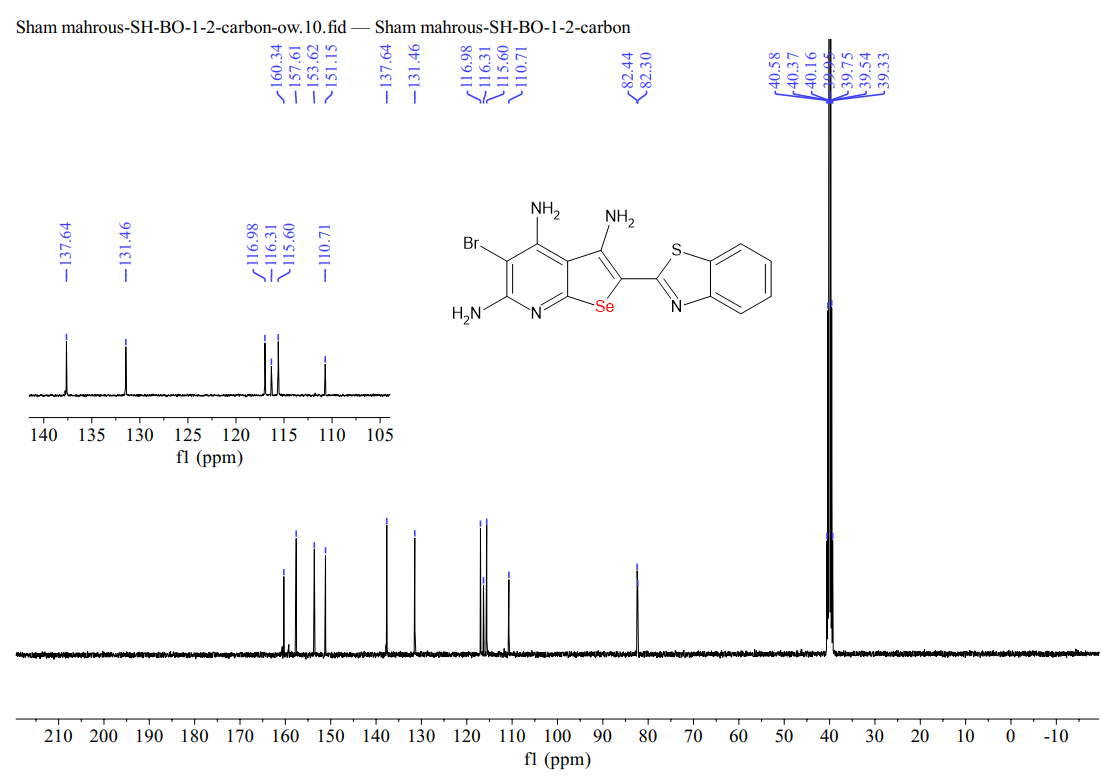


- 1. **IR, ^1^H (D_2_O) and ^13^C NMR Spectra of 11:**


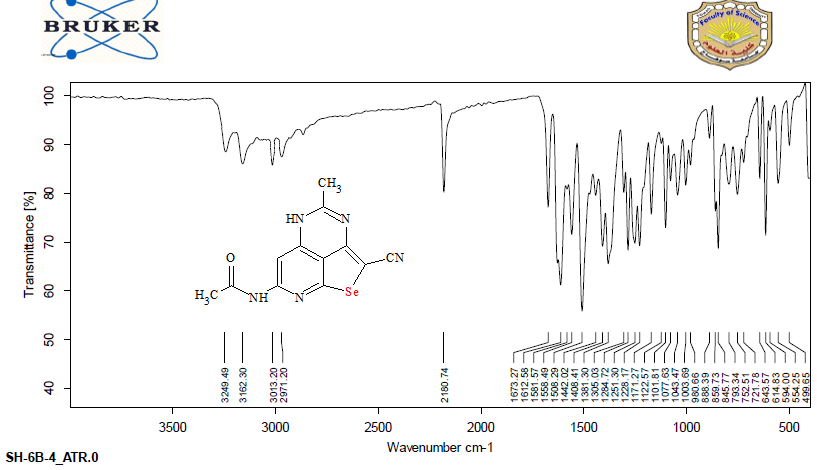


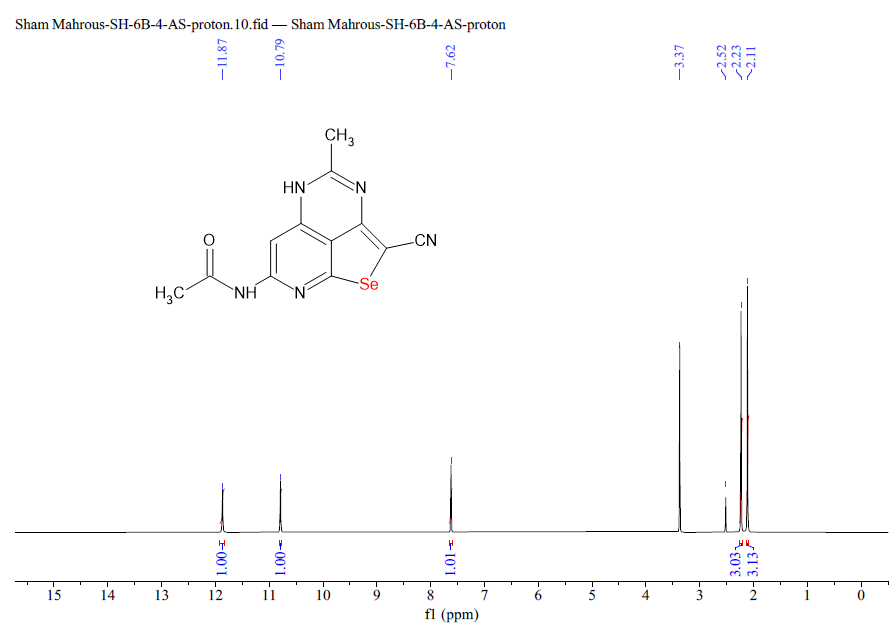


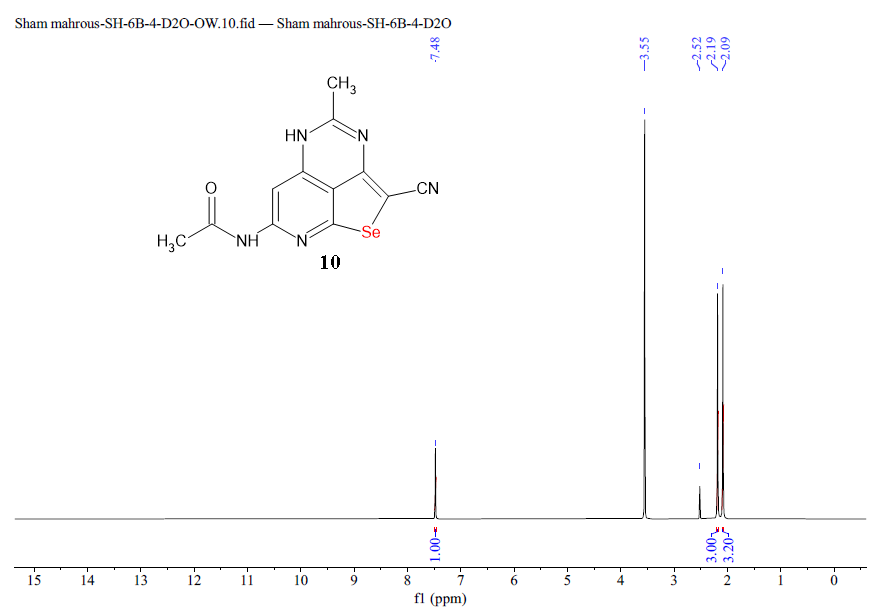


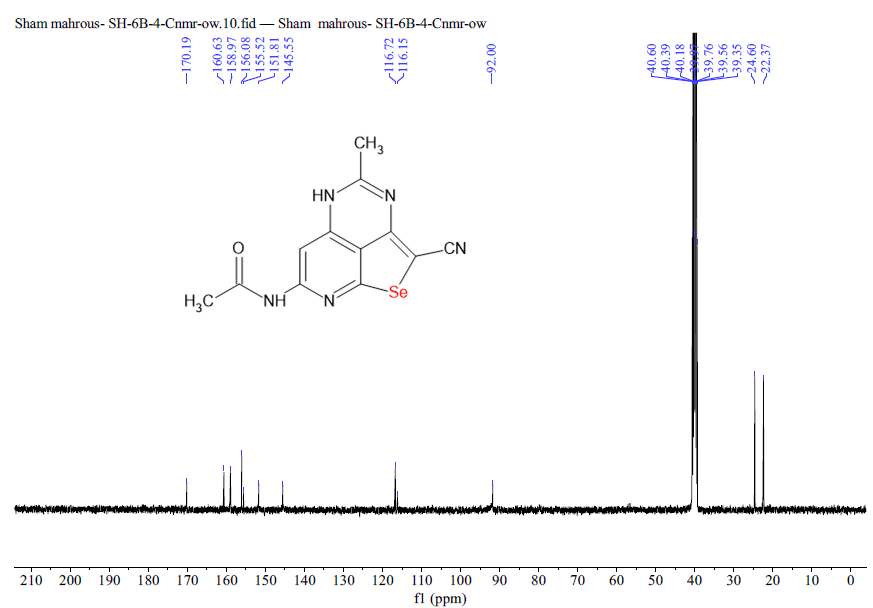


- 1. **IR, ^1^H and ^13^C NMR Spectra of 12a**:


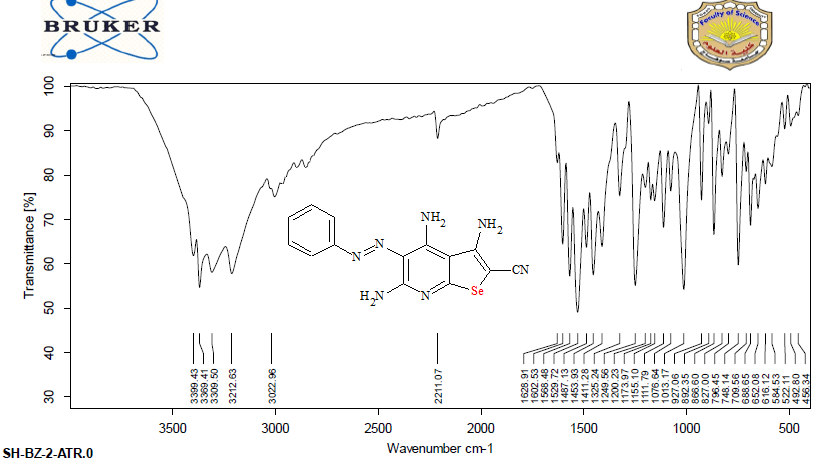


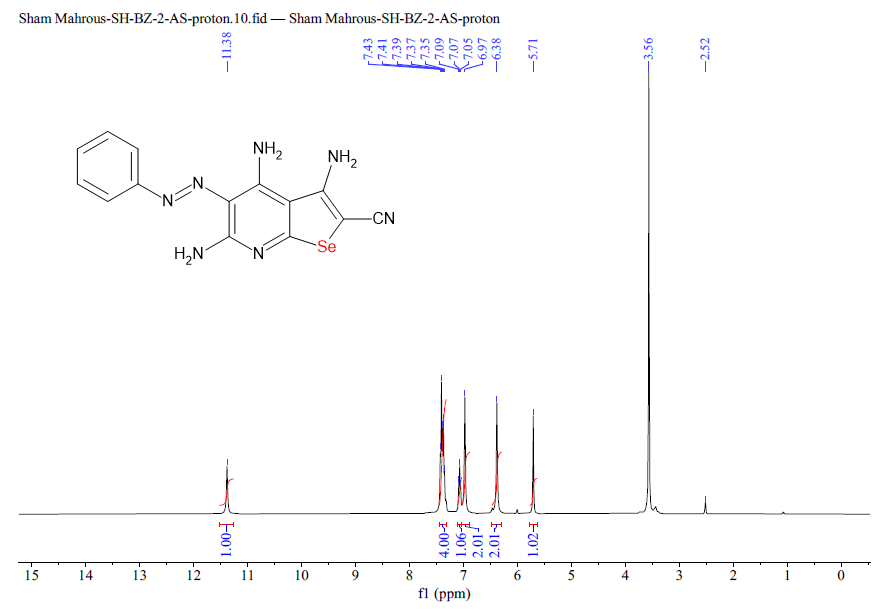


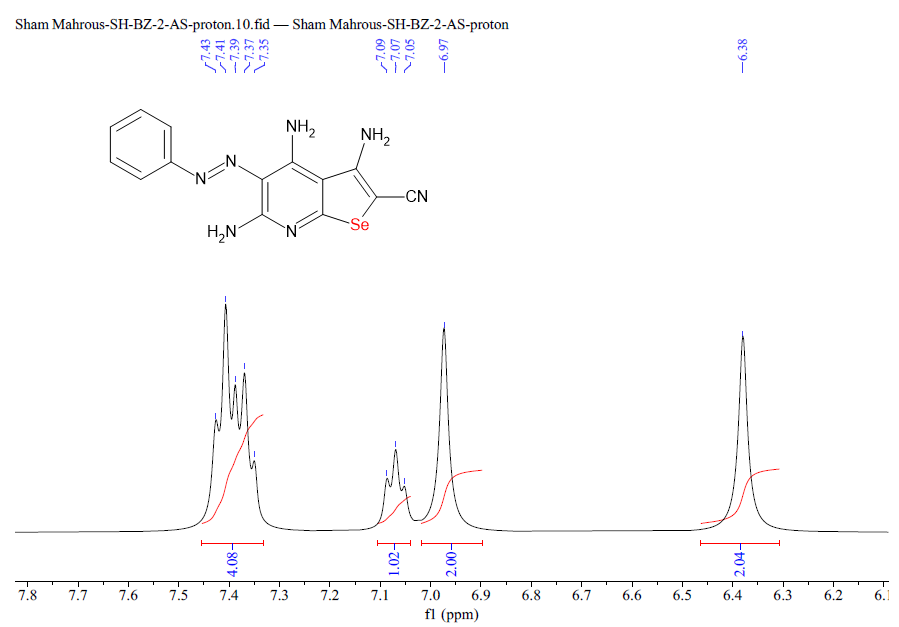


**
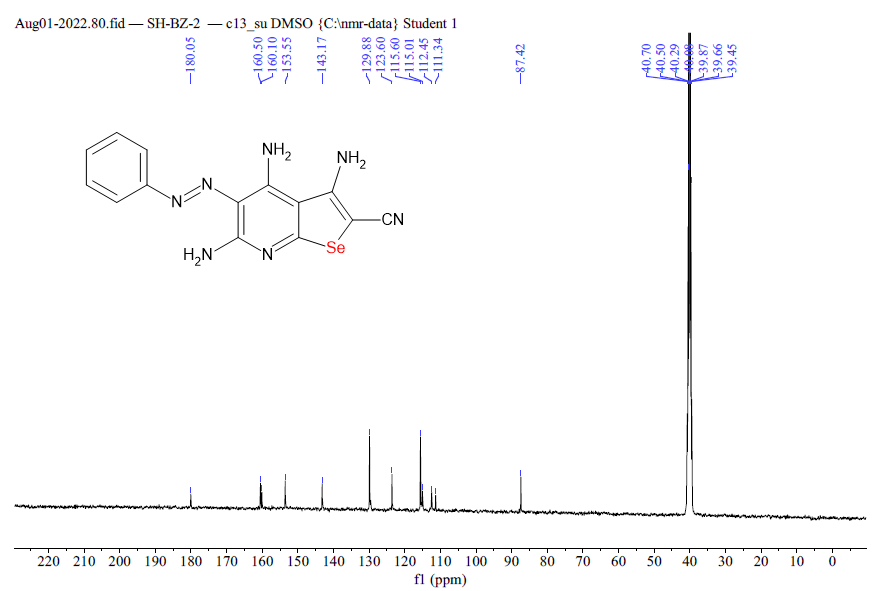
**

- 1. **IR, ^1^H and ^13^C NMR Spectra of 12b**:

**
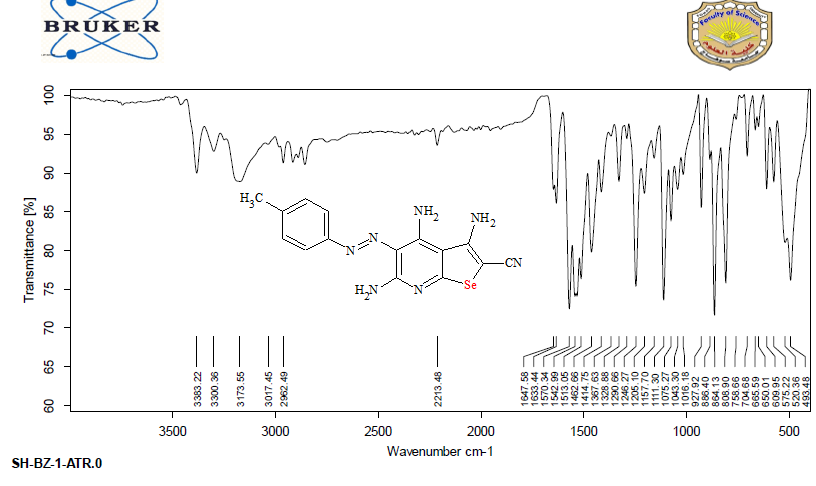
**


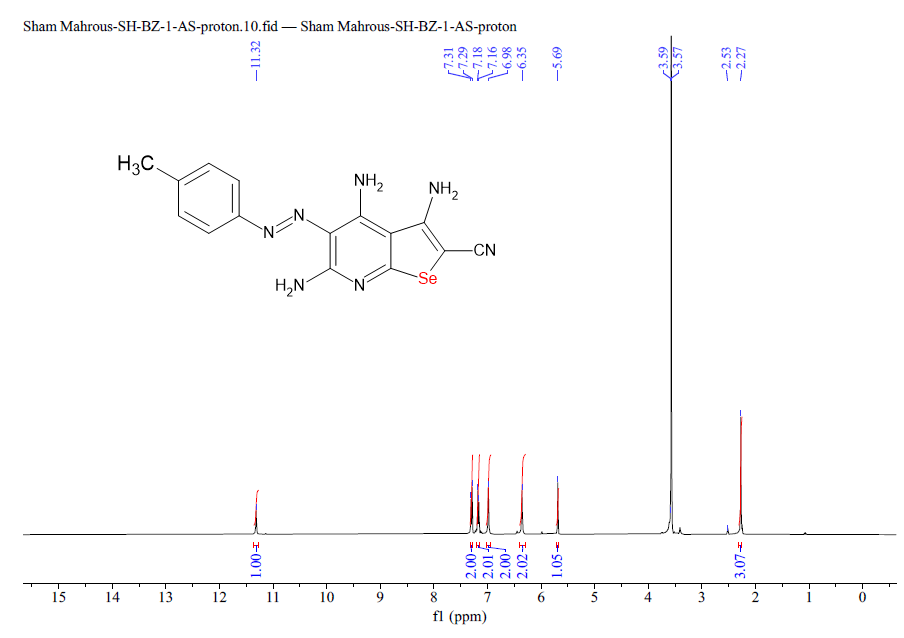


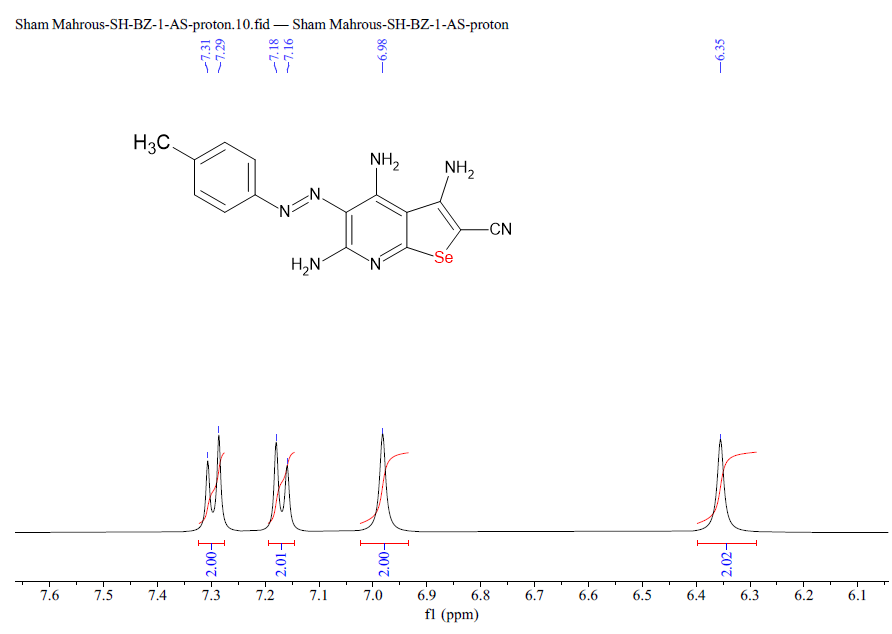


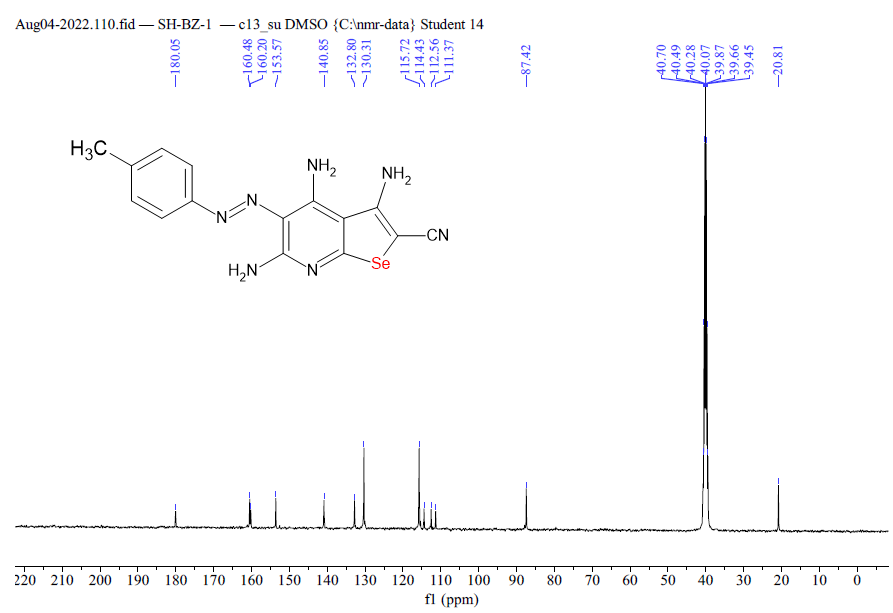


- 1. **IR, ^1^H and ^13^C NMR Spectra of 12c:**

**^
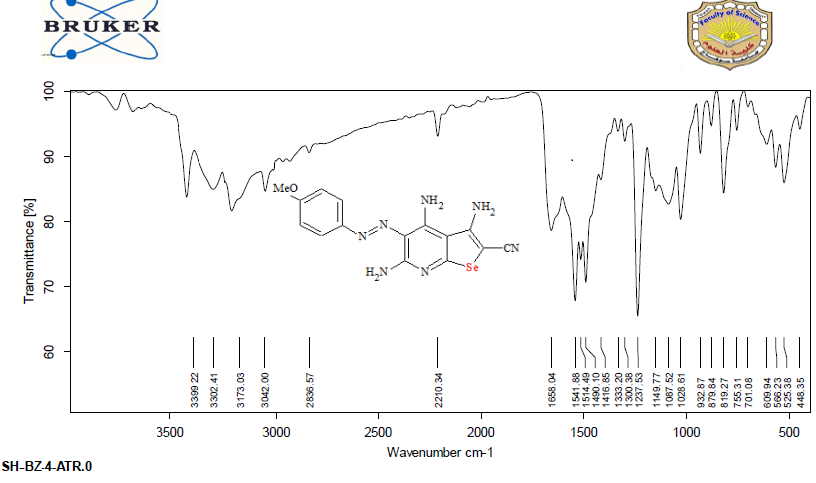
^**


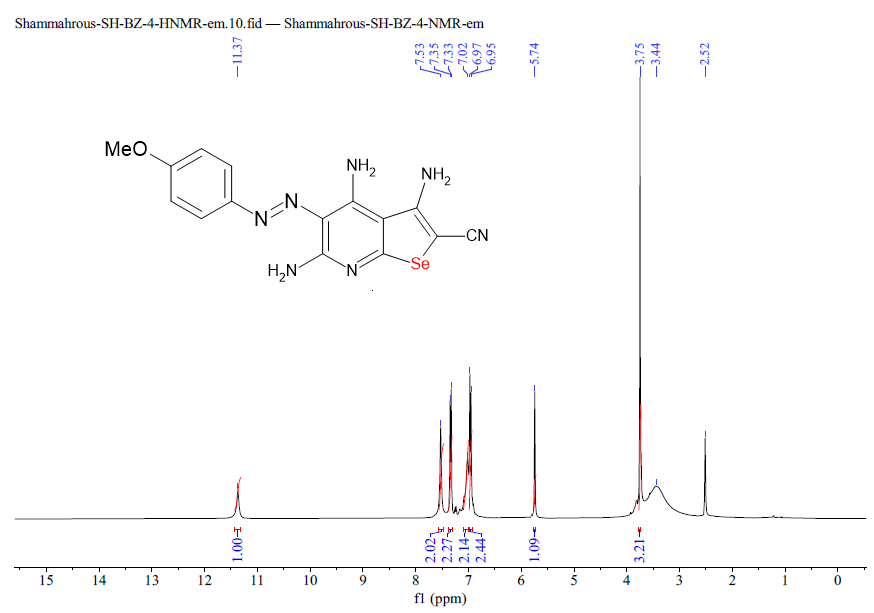


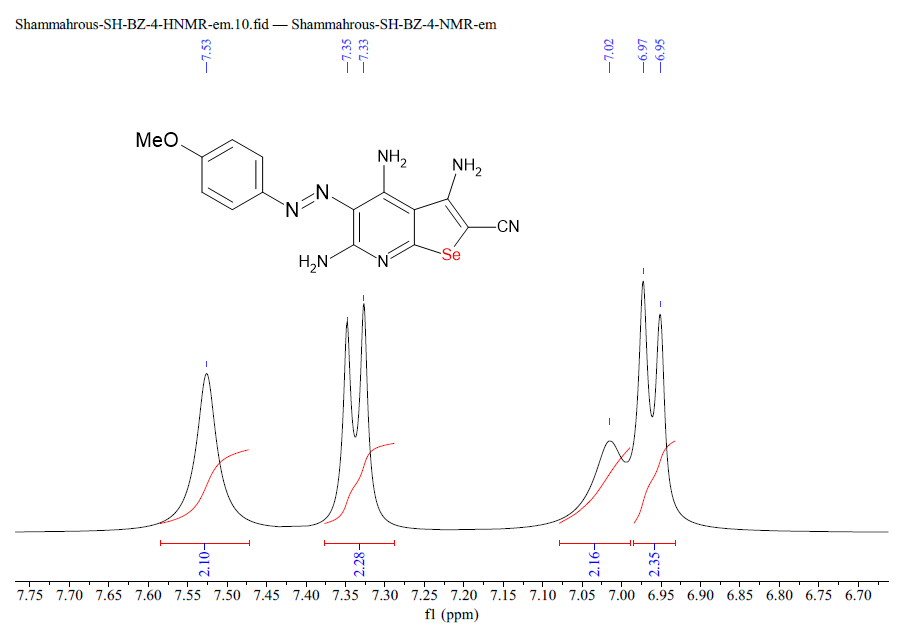


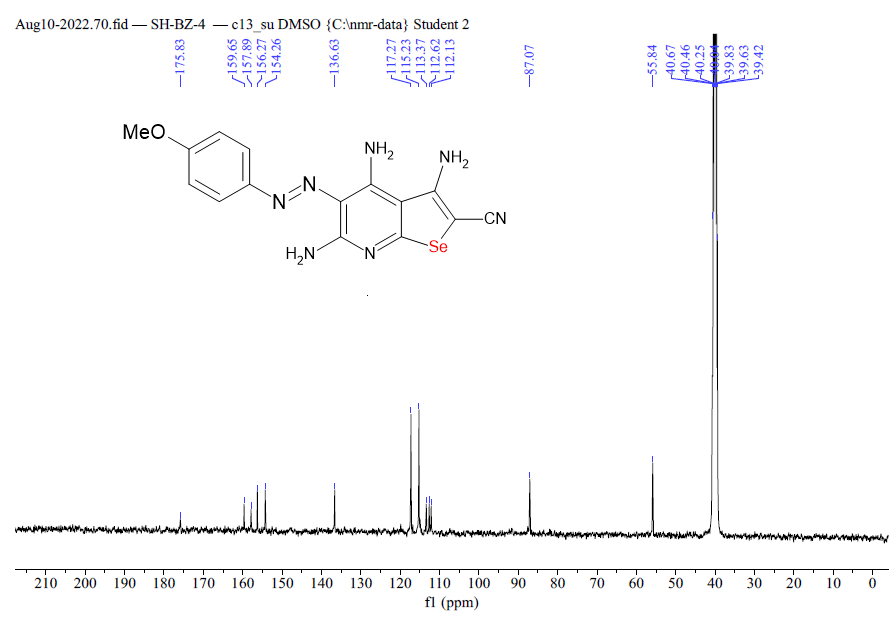


- 1. **IR, ^1^H and ^13^C NMR Spectra of 12d**:

**
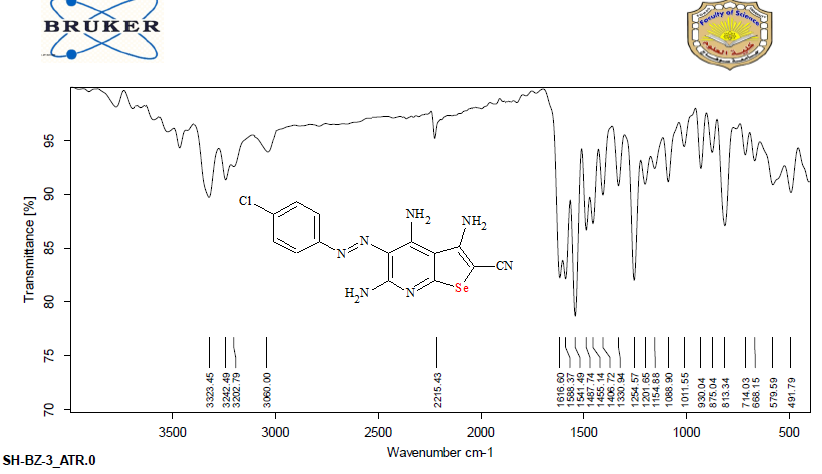
**


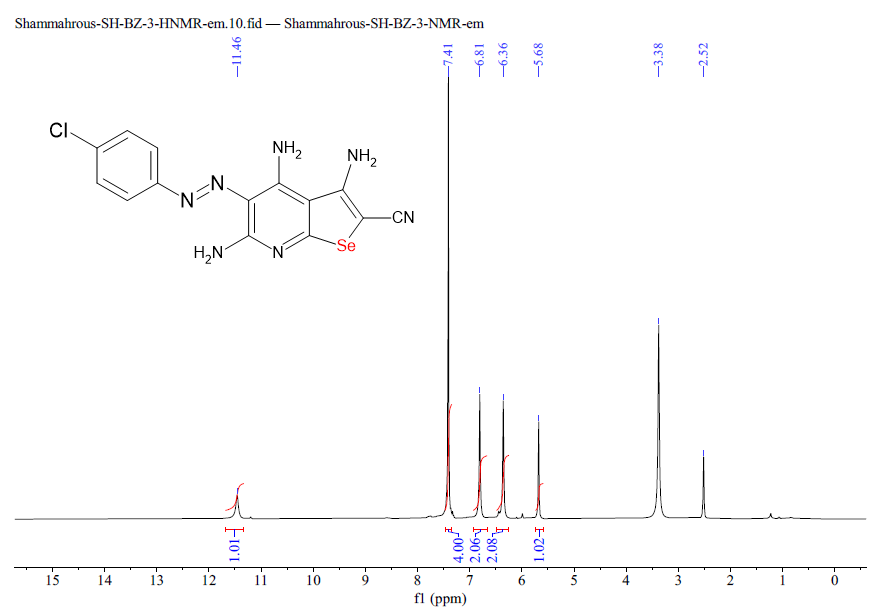


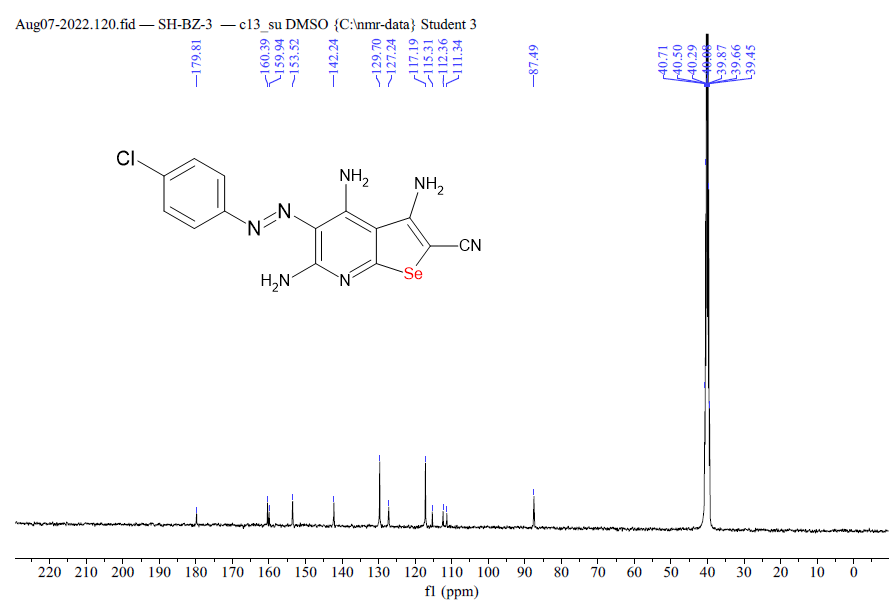

Supplement: Supplementary file 1 — Supplementary file1 (DOCX 2940 KB) [file 11030_2024_10872_MOESM1_ESM.docx]
